# Mentalization, Emotional Dysregulation, and Emotion-Related Impulsivity in Young Adults with Borderline Personality Disorder

**DOI:** 10.3390/brainsci16080801

**Published:** 2026-07-29

**Authors:** Anaïs Mungo, Marie Delhaye, Matthieu Hein

**Affiliations:** 1 Department of Psychiatry, CH Le Domaine-ULB, Université Libre de Bruxelles (ULB), 1420 Braine l’Alleud, Belgium; anais.mungo@ulb.be; 2 Unit 2, Department of Psychiatry, La Ramée, Epsylon, 1180 Brussels, Belgium; marie.delhaye@umons.ac.be; 3 Laboratoire de Psychologie Médicale et Addictologie (ULB312), CHU Brugmann, Université Libre de Bruxelles (ULB), 1020 Brussels, Belgium

**Keywords:** borderline personality disorder, mentalization, reflective functioning, emotional dysregulation, impulsivity, young adult

## Abstract

**Highlights:**

**What are the main findings?**

- Young adults with borderline personality disorder (BPD) reported greater uncertainty regarding mental states (RFQu) than non-clinical controls, even after adjustment for anxiety–depressive symptoms and other clinical factors.
- Uncertainty regarding mental states was associated with emotion-related impulsivity, particularly negative urgency, beyond the contribution of alexithymia and anxiety–depressive symptomatology.

**What are the implications of the main findings?**

- Mentalizing difficulties may represent an additional factor associated with emotion-related impulsivity in emerging BPD.
- Assessing and targeting mentalization may help identify vulnerable young individuals and inform early interventions aimed at reducing impulsive behaviors in BPD.

**Abstract:**

**Background**: Borderline personality disorder (BPD) is characterized by emotional dysregulation and emotion-related impulsivity, particularly during late adolescence and early adulthood. Although mentalizing difficulties have been proposed as an important mechanism underlying borderline psychopathology, their contribution to emotion-related impulsivity beyond alexithymia and anxiety–depressive symptoms remains unclear. This study examined the relationships among mentalizing difficulties, emotional dysregulation, and emotion-related impulsivity in young individuals with BPD. **Methods**: A total of 166 participants aged 16–25 years were recruited in French-speaking Belgium, including 45 individuals with DSM-5 BPD and 121 non-clinical controls. Mentalization was assessed using the Reflective Functioning Questionnaire (RFQ-8), emotional dysregulation using the Difficulties in Emotion Regulation Scale (DERS), impulsivity using the UPPS-P Impulsive Behavior Scale, and alexithymia using the Toronto Alexithymia Scale (TAS-20). Group comparisons, quantile regressions, Spearman correlations, and adjusted multiple linear regressions were performed. **Results**: Participants with BPD reported significantly higher uncertainty regarding mental states (RFQu) than controls, and this difference remained significant after adjustment for clinical covariates. RFQu was associated with overall emotional dysregulation, particularly difficulties in behavioral control under distress, and with emotion-related impulsivity, especially negative urgency. Associations between RFQu and negative urgency remained significant after controlling for alexithymia, depressive symptoms, trait anxiety, and borderline status. Corresponding associations with positive urgency did not reach statistical significance. **Conclusions**: Mentalizing difficulties may account for additional variance in emotion-related impulsivity beyond alexithymia and general psychopathological distress. These findings support a developmental, mechanism-focused understanding of emerging BPD and highlight mentalization as a potential target for early intervention and future longitudinal research.

## 1. Introduction

### 1.1. Emotional Dysregulation as a Central and Transdiagnostic Process

Emotional dysregulation is now considered a major transdiagnostic dimension involved in numerous psychiatric disorders. In their multidimensional model, Gratz and Roemer define emotion regulation as the ability to be aware of one’s emotions, to understand and accept them, to maintain goal-directed behaviors despite emotional distress, to control impulsive behaviors, and to implement regulation strategies that are appropriate to the context. Emotional dysregulation therefore refers to difficulties in one or more of these dimensions [1].

Contemporary models conceptualize emotional dysregulation as a central vulnerability factor associated with affective instability, impulsivity, and self-damaging behaviors [2]. Overall levels of emotional dysregulation appear to be closely associated with anxiety, depressive, and borderline symptoms, highlighting its cross-cutting role in psychopathological expression [3].

Emotional dysregulation is also strongly associated with impulsive behaviors. Certain behaviors, such as non-suicidal self-injury, substance use, or risky sexual behaviors, may be understood as attempts to obtain rapid relief from emotional states perceived as intolerable [2,4].

These issues assume particular importance during late adolescence and early adulthood, a developmental period characterized by the still incomplete maturation of the neural networks involved in emotion regulation and behavioral control. The earlier maturation of emotional reactivity systems relative to prefrontal control systems is thought to confer greater vulnerability to emotional hyperreactivity and impulsive behaviors [3,5].

### 1.2. Borderline Personality Disorder as a Clinical Model of Emotional Dysregulation

Borderline personality disorder (BPD) is a severe psychiatric disorder characterized by marked instability in affect, interpersonal relationships, self-image, and impulse control [6,7]. Contemporary models conceptualize emotional dysregulation as one of the core mechanisms underlying the disorder, influencing the affective, behavioral, and interpersonal manifestations of BPD [2].

This conceptualization is notably supported by Linehan’s biosocial model, according to which BPD results from the interaction between a constitutional emotional vulnerability and a chronically invalidating environment [8]. Within this framework, impulsive and self-damaging behaviors are often understood as attempts to modulate particularly intense emotional states that are difficult to regulate [2,8].

Recent studies conducted among young adults indicate that individuals with BPD report marked difficulties in behavioral control under conditions of emotional distress, emotional awareness, emotional clarity, and access to adaptive emotion regulation strategies [3]. Furthermore, the impulsivity observed in BPD appears to be primarily emotion-based, with negative urgency and positive urgency emerging as particularly robust markers of the disorder in young adults [9].

### 1.3. Beyond Emotional Dysregulation: Understanding the Mechanisms Underlying Borderline Impulsivity

Although the central role of emotional dysregulation in BPD is now widely recognized, the mechanisms explaining why some individuals respond to their emotions through impulsive behaviors remain only partially understood. This impulsivity cannot be reduced to a mere generalized deficit in inhibitory control, but appears embedded within a complex emotional dynamic that is highly dependent on intense affective states [2,9].

Contemporary models suggest that the impulsive behaviors observed in BPD may result not only from emotional hyperreactivity but also from more fundamental difficulties in processing emotional experiences and mental states [10,11]. This perspective has led to growing interest in various processes involved in the understanding and regulation of emotions, among which alexithymia occupies a prominent place in the literature.

However, several authors have also proposed that mentalizing capacities may help explain how emotional states are identified, understood, and integrated within interpersonal situations [12]. From this perspective, mentalization appears less as a mechanism specific to BPD than as a process capable of integrating the affective, interpersonal, and behavioral dimensions of the disorder. Accordingly, the interplay between emotional dysregulation, impulsivity, and mentalization has assumed an increasingly important role in contemporary models of BPD, although no consensus has yet emerged regarding the precise nature of their relationships.

Thus, although emotional dysregulation appears to be a fundamental mechanism in BPD, investigating the processes through which individuals understand and make sense of their emotional experiences may contribute to a better understanding of the emergence of impulsive behaviors observed in this disorder. This perspective provides the rationale for the present study’s focus on mentalization.

### 1.4. Alexithymia: A Relevant but Insufficient Construct to Explain Borderline Impulsivity

Alexithymia, defined as a difficulty in identifying, differentiating, and verbalizing emotions, has been extensively studied in BPD [13,14]. Several studies have shown that individuals with BPD exhibit particularly high levels of alexithymia, especially with regard to the dimensions of difficulty identifying feelings and difficulty describing feelings [15,16,17,18]. These difficulties appear to be closely associated with emotional dysregulation, attachment disturbances, and early traumatic experiences that are frequently observed in BPD [19,20,21]. Several authors have therefore suggested that alexithymia may constitute a vulnerability factor that promotes poorly differentiated emotional responses that are difficult to modulate [22].

Within this framework, several studies have proposed that difficulties in emotional identification may indirectly contribute to the impulsive behaviors observed in BPD. Meaney et al. notably demonstrated that alexithymia, rumination, and emotional dysregulation contribute to the occurrence of dysregulated behaviors such as self-injury, substance use, and aggression [23]. Similarly, Velotti et al. suggested that difficulties in identifying and processing affective states may foster impulsive responses when emotions become difficult to tolerate [22]. The work of Gaher et al. further showed that difficulty identifying feelings mediates the relationship between childhood maltreatment and negative urgency, supporting the notion that alexithymia may serve as an intermediary mechanism linking emotional vulnerability to emotion-based impulsivity [24].

Nevertheless, despite the associations reported in the literature, findings remain heterogeneous among individuals with BPD. In our previous study comparing young adults with BPD to non-clinical controls, participants with BPD exhibited significantly higher levels of alexithymia than controls [25]. Moreover, in the overall sample, alexithymia was associated with several dimensions of emotion-related impulsivity, supporting the hypothesis that difficulties in identifying and verbalizing emotions may be linked to a greater dispositional tendency to act impulsively in emotionally intense contexts. However, the associations observed between alexithymia and impulsivity in the overall sample were attenuated and no longer reached statistical significance when analyses were restricted to the BPD subgroup. Given the modest sample size and the clinical severity of this subgroup, these findings warrant cautious interpretation [25]. Taken together, these observations suggest that the contribution of alexithymic difficulties to emotion-related impulsivity within BPD may be more complex than initially expected and warrant the investigation of additional processes, including difficulties in mentalization.

They also invite consideration of other processes involved in the understanding and interpretation of mental states, the importance of which has been emphasized in several studies of borderline functioning [11]. This limitation has led researchers to focus on broader models of socio-emotional functioning that account not only for the identification of emotions but also for the understanding and interpretation of one’s own and others’ mental states. Among these models, mentalization now occupies a central position.

### 1.5. Mentalization and Borderline Personality Disorder

Mentalization refers to the capacity to understand and interpret one’s own behavior and that of others in terms of underlying mental states, such as thoughts, emotions, intentions, desires, or beliefs [26,27]. This capacity enables individuals to make sense of interpersonal experiences and to regulate emotional responses by considering both internal psychological realities and environmental constraints. Developmental models of mentalization suggest that it may play an important role in affect regulation, identity formation, and interpersonal functioning [12,27].

According to these models, this capacity develops progressively within the context of early attachment relationships. The development of an adequate reflective function is thought to depend on caregivers’ ability to recognize, mirror, and respond appropriately to the child’s emotional states. Through repeated experiences of marked and contingent affect mirroring, children gradually learn to identify their internal experiences as meaningful mental states and to differentiate psychic reality from external reality [27,28,29]. Secure attachment relationships thus constitute an essential foundation for the development of a coherent sense of self and adaptive emotion regulation capacities [26,27].

Conversely, experiences of neglect, trauma, or attachment insecurity may compromise the development of mentalizing capacities and promote the emergence of difficulties in understanding one’s own and others’ mental states, particularly in situations involving heightened emotional activation or interpersonal stress [10,12]. In the mentalization-based model of BPD, these disturbances constitute a central vulnerability contributing to the emotional instability, interpersonal difficulties, identity disturbances, and impulsive behaviors characteristic of the disorder [10,30,31].

Contemporary research nevertheless emphasizes that the mentalizing difficulties observed in BPD do not necessarily reflect a stable and global deficit, but rather emerge under specific emotional and interpersonal conditions [10,32]. Sharp et al. demonstrated that adolescents exhibiting higher levels of borderline traits simultaneously displayed greater difficulties in emotion regulation and disturbances in the processing of mental states [33]. Sharp and Vanwoerden further proposed that these disturbances frequently take the form of hypermentalizing, characterized by the excessive or inaccurate attribution of mental states to oneself or others [11].

Recent empirical evidence also suggests that the mentalizing difficulties observed in BPD primarily involve complex processes of reasoning about mental states rather than simple emotional decoding. In a meta-analysis of 17 studies, Németh et al. showed that individuals with BPD exhibit significant impairments in theory of mind, particularly on tasks requiring the integration of complex contextual and interpersonal information. The authors suggested that the observed alterations primarily affect reasoning about mental states, whereas basic emotion recognition abilities appear to be relatively less impaired [34].

The development of the Reflective Functioning Questionnaire (RFQ) has enabled the assessment of mentalizing capacities through a self-report instrument [35]. This questionnaire distinguishes between two complementary dimensions: certainty about mental states (RFQc) and uncertainty about mental states (RFQu). Although excessive certainty was initially proposed as a marker of hypermentalizing, several recent studies have highlighted psychometric limitations associated with this dimension. In contrast, elevated RFQu scores, reflecting greater uncertainty regarding mental states and generally interpreted as a form of hypomentalizing, appear to constitute the most robust indicator of mentalizing difficulties assessed by the RFQ [36]. The French validation of the RFQ showed that higher levels of uncertainty regarding mental states were associated with more pronounced borderline traits, greater alexithymia, increased general psychopathology, and higher levels of non-suicidal self-injurious behaviors [37].

More recently, the development of multidimensional instruments such as the Mentalization Scale (MentS) has enabled a more refined assessment of mentalization by distinguishing between self-mentalizing, other-mentalizing, and motivation to mentalize. The recent French validation of this instrument confirmed satisfactory psychometric properties in clinical samples that notably included individuals with BPD. Mentalization scores were associated with several indicators of psychopathology and psychological functioning, supporting the clinical relevance of this measure [38]. Taken together, these findings reinforce the notion that mentalization constitutes a central dimension of borderline functioning.

### 1.6. Mentalization, Emotional Dysregulation, and Impulsivity in BPD

A growing body of research suggests that mentalizing difficulties contribute to the clinical manifestations of BPD through their impact on emotion regulation processes. These models conceptualize mentalization as an essential mechanism enabling individuals to identify, understand, and modulate emotional experiences. When this capacity is impaired, emotions become more difficult to interpret and regulate, thereby promoting the emergence of maladaptive behavioral responses [32,39].

Furthermore, these models no longer conceptualize the mentalizing difficulties observed in BPD as reflecting a stable and global deficit in reflective capacities. Rather, they suggest that such difficulties emerge primarily in contexts characterized by heightened emotional and interpersonal activation. Accordingly, mentalizing capacities may remain relatively preserved in emotionally neutral situations but become transiently disorganized during experiences of rejection, abandonment, or intense affective distress. This context-dependent loss of mentalization may, in turn, compromise the understanding and regulation of emotional experiences, thereby facilitating the subsequent emergence of emotionally driven impulsive behaviors [11,12,32].

Several empirical studies support this perspective. Kahya and Munguldar demonstrated, in a large sample of adolescents drawn from the general population, that difficulties in emotion regulation partially mediated the relationship between reflective functioning, as assessed by the RFQ, and borderline symptoms. More specifically, lack of emotional clarity, difficulties in impulse control, and limited access to adaptive emotion regulation strategies contributed significantly to explaining the association between mentalizing difficulties and borderline symptomatology [40]. Consistent with these findings, Euler et al. reported that hypomentalizing was not directly associated with interpersonal difficulties among individuals with BPD but exerted a significant indirect effect through emotional dysregulation and attentional impulsivity. These findings suggest that mentalizing difficulties may be linked to the clinical manifestations of BPD through affective and behavioral processes, rather than exerting a direct influence [41].

The associations between mentalization and borderline psychopathology also appear to be embedded within a developmental framework. Quek et al. found that reflective functioning mediated the relationship between experiences of emotional maltreatment and borderline traits in adolescents, suggesting that adverse early relational experiences may increase vulnerability to BPD by disrupting the development of mentalizing capacities [42]. These findings are consistent with those of Perroud et al., who observed that adults with BPD reported greater mentalizing difficulties compared with both healthy controls and individuals with attention-deficit/hyperactivity disorder (ADHD) [43].

Taken together, mentalizing difficulties may constitute an important mechanism linking early developmental experiences, emotional dysregulation, and impulsive behaviors in BPD, a link particularly relevant during young adulthood, when emotion regulation, identity, and reflective abilities continue to mature.

### 1.7. Study Objectives and Hypotheses

Building upon contemporary models of mentalization and recent findings concerning emotional dysregulation and impulsivity in BPD, the present study aims to improve our understanding of the role of mentalizing capacities in the emotional difficulties and impulsive tendencies reported by young adults with BPD. More specifically, this study investigates the relationships among reflective functioning, emotional dysregulation, and traits of emotion-related impulsivity within an integrative and developmental framework focused on the underlying psychopathological mechanisms.

The first objective of this study is to compare self-reported mentalizing capacities between young adults with BPD and control participants using the dimensions of the Reflective Functioning Questionnaire (RFQ-8). Consistent with previous research, particular attention will be paid to the dimension of uncertainty about mental states (RFQu), generally associated with hypomentalizing, as well as to the dimension of certainty about mental states (RFQc). The latter was initially proposed as an indirect indicator of certain forms of hypermentalizing, although this interpretation is currently debated on both theoretical and psychometric grounds [11,36]. Accordingly, the RFQc dimension will be considered cautiously and primarily in an exploratory manner. Therefore, the present study will focus principally on RFQu as the central indicator of mentalizing difficulties in BPD.

The second objective is to examine the associations among mentalizing capacities, emotional dysregulation, and multidimensional impulsivity. More specifically, the relationships between the dimensions of the RFQ-8, the subscales of the Difficulties in Emotion Regulation Scale (DERS), and the dimensions of the UPPS-P will be investigated.

The third objective, exploratory in nature, is to determine whether mentalizing difficulties are associated with emotion-related impulsivity beyond other psychopathological variables known to be involved in BPD. More specifically, this study will examine whether hypomentalizing, assessed through the RFQu dimension, remains associated with the emotional dimensions of impulsivity after controlling for alexithymia, anxiety symptoms, and depressive symptoms. This question appears particularly important considering the findings from our previous study, which showed that alexithymia was significantly higher among young adults with BPD, but that its associations with impulsivity became substantially weaker and non-significant when analyses were restricted to the BPD group alone [25].

Few studies have simultaneously examined the relationships among self-reported mentalization, emotional dysregulation, and emotion-related impulsivity in young adults with BPD while implicitly comparing the specific contribution of mentalization with that of alexithymia. Most existing studies have focused either on emotion regulation difficulties or on mentalizing capacities without fully integrating these dimensions within a single psychopathological model. Accordingly, the present study aims to contribute to a better understanding of the psychological mechanisms underlying borderline emotion-related impulsivity by examining more specifically the role of uncertainty regarding mental states.

Within this framework, we hypothesize that higher levels of uncertainty regarding mental states (RFQu) will be associated with greater difficulties in emotion regulation, particularly with respect to emotional clarity (DERS Clarity) and behavioral control under emotional distress (DERS Impulse), two dimensions identified as especially important in the associations between mentalization and borderline symptomatology [40]. We further hypothesize that elevated RFQu scores will be associated with negative urgency. Given the limited literature regarding positive urgency, associations with this dimension will be examined on an exploratory basis. Finally, we hypothesize that these associations will remain significant after controlling for alexithymia and anxiety–depressive symptomatology. Based on our previous findings and the emerging literature on mentalization in BPD, we hypothesize that uncertainty regarding mental states may account for additional variance in emotion-related impulsivity after accounting for alexithymia and anxiety–depressive symptomatology [25]. More broadly, this study is grounded in a transdiagnostic, developmental, and mechanism-focused approach. It is based on the theoretical framework proposing that the mentalizing difficulties observed in BPD emerge particularly in contexts of heightened emotional and interpersonal activation, thereby secondarily promoting difficulties in emotion regulation and emotionally induced impulsive behaviors. Although this context-dependent loss of mentalization is not directly assessed in the present study, it constitutes the theoretical model from which the hypotheses under investigation are derived.

## 2. Materials and Methods

### 2.1. Methodological Framework and Cohort Continuity

This study is part of a broader research program devoted to the emotional and interpersonal processes involved in BPD among older adolescents and young adults aged 16 to 25 years. Previous investigations conducted within this cohort have examined various aspects of the psychological mechanisms associated with BPD, including multidimensional impulsivity profiles assessed using the UPPS-P model, the relationships between emotional dysregulation and impulsivity, and the associations between alexithymia and dimensions of emotion-related impulsivity [3,9,25].

In continuity with this work, the present study aims to explore mentalizing capacities as a process potentially involved in the manifestations of impulsivity and emotional dysregulation observed in young individuals with BPD. More specifically, the study focuses on difficulties in understanding and interpreting mental states, assessed using the RFQ, and their associations with the emotional dimensions of impulsivity and emotional dysregulation.

The study is based on a previously established clinical and non-clinical cohort developed as part of the earlier investigations, thereby ensuring consistency in recruitment procedures, diagnostic assessment, and administration of psychometric instruments. This methodological approach promotes coherence across comparisons conducted within the different research axes of the program while addressing a specific research question centered on mentalization.

The analyses presented in this study do not constitute a replication of the previous studies but rather a theoretical and methodological extension of them. The present protocol places mentalization at the center of the investigation to achieve a better understanding of the mechanisms underlying emotion-related impulsivity in BPD during this critical developmental period.

### 2.2. Participants

Participants were recruited between January 2021 and October 2023 in French-speaking Belgium, within both clinical and community settings, to establish a comparative sample composed of young individuals with BPD and participants without known psychiatric disorders.

#### 2.2.1. BPD Group

The clinical group consisted of older adolescents and young adults receiving care within adolescent psychiatry and adult psychiatry departments of a Belgian university hospital, either through inpatient services or outpatient follow-up programs.

The inclusion criteria were as follows:

- being between 16 and 25 years of age;
- having sufficient proficiency in spoken and written French;
- meeting DSM-5 criteria for BPD, with the diagnosis confirmed both through the clinical evaluation conducted by the treating psychiatrist and through administration of the Diagnostic Interview for Borderlines–Revised (DIB-R).

Inter-rater reliability for the DIB-R was not specifically assessed in the present study. However, the diagnosis was established through a dual procedure combining clinical evaluation by the treating psychiatrist and confirmation using the DIB-R administered by a trained researcher. Previous studies have demonstrated satisfactory inter-rater reliability of the DIB-R.

Participants were excluded in cases of:

- inability to understand or complete the assessment instruments;
- documented intellectual disability;
- a history of, or current, psychotic disorder;
- autism spectrum disorder;
- severe medical conditions likely to significantly affect overall functioning or short-term prognosis, including certain progressive neurological disorders, oncological diseases, or advanced organ failure.

No additional selection criteria were applied regarding symptom severity, psychiatric comorbidities, or duration of therapeutic care. This approach was intended to preserve the clinical representativeness of BPD as encountered in routine psychiatric practice among young adults.

#### 2.2.2. Non-Clinical Group

The non-clinical comparison group was recruited from a variety of non-psychiatric settings, including secondary schools in Wallonia and Brussels, several faculties of the Université libre de Bruxelles, and information campaigns conducted within the university hospital.

The inclusion criteria were:

- being between 16 and 25 years of age;
- having sufficient proficiency in the French language.

The exclusion criteria were:

- any diagnosed psychiatric disorder;
- current engagement in specialized psychiatric or psychological treatment;
- current use of psychotropic medication;
- the presence of severe somatic conditions comparable to those excluded in the clinical group.

Following verification of eligibility criteria and exclusion of participants who did not meet the required conditions, the control sample consisted of young individuals without known psychiatric disorders, thereby constituting a non-clinical comparison group appropriate to the objectives of the study.

### 2.3. Procedure and Data Collection

Prior to participation, all participants received comprehensive information regarding the scientific objectives of the study, the assessment procedures, and the confidentiality conditions governing the collected data. Written informed consent was obtained from each participant before inclusion in the study. For participants under the age of 18 years, written consent from a parent or legal guardian was also required, in accordance with ethical recommendations applicable to research involving adolescents.

Assessments were conducted in French using self-report questionnaires administered in settings supervised by a trained member of the research team, either within participating clinical services or in the schools and universities involved in recruitment.

Assignment to the clinical group required the presence of borderline personality disorder meeting DSM-5 diagnostic criteria. The diagnosis relied on a dual assessment procedure combining the clinical expertise of the treating psychiatrist with administration of the DIB-R, thereby strengthening the diagnostic validity of the BPD group.

Sociodemographic and clinical data were collected in a standardized manner from all participants. Recorded variables included age, gender, student status, and educational attainment at the time of inclusion. Participants also reported their personal medical, surgical, and psychiatric histories, as well as the presence of any current psychiatric comorbidities.

Substance use habits were also assessed, including the consumption of alcohol and illicit psychoactive substances. Family psychiatric history was documented for exploratory purposes in order to account for potential factors of psychopathological vulnerability. Regarding suicidal behaviors, participants were asked about any history of suicide attempts, coded dichotomously (presence/absence), while the total number of suicide attempts was recorded as a continuous variable. Finally, information concerning psychotherapeutic and pharmacological treatment was collected, including past or current psychological follow-up and the use of psychotropic medication at the time of assessment.

Depressive symptoms were assessed using the Beck Depression Inventory—Second Edition (BDI-II), a self-report questionnaire comprising 21 items rated on a scale from 0 to 3. This instrument explores various dimensions of depressive symptomatology, including affective, cognitive, motivational, and somatic aspects such as depressed mood, negative cognitions, feelings of worthlessness, fatigue, sleep disturbances, and changes in appetite. The total score provides an estimate of the overall severity of depressive symptoms according to severity thresholds commonly used in the clinical literature (minimal, mild, moderate, or severe) [44].

Trait anxiety was measured using the Trait subscale of the State–Trait Anxiety Inventory (STAI-T), which consists of 20 items assessing the stable tendency to perceive situations as threatening and to respond with anxious emotional arousal. The STAI-T is widely used in clinical research and demonstrates robust psychometric properties, including excellent internal consistency and good convergent validity in adolescent and adult psychiatric populations [45].

Impulsivity was assessed using the French short version of the UPPS-P Impulsive Behavior Scale, a self-report instrument providing a multidimensional approach to impulsivity. This scale comprises 20 items distributed across five dimensions: negative urgency, positive urgency, lack of premeditation, lack of perseverance, and sensation seeking. The positive and negative urgency dimensions specifically assess the tendency to act impulsively in emotionally intense positive and negative contexts. The French short version of the UPPS-P demonstrates satisfactory psychometric properties, including adequate internal consistency across all subscales and good temporal stability [46].

Emotional dysregulation was assessed using the Difficulties in Emotion Regulation Scale (DERS), a 36-item self-report questionnaire assessing habitual (rather than transient) difficulties in emotion regulation across six dimensions: Nonacceptance (difficulty accepting negative emotions), Goals (difficulty maintaining goal-directed behavior under emotional distress), Impulse (difficulty with behavioral control under emotional distress), Awareness (reduced attention to one’s emotional states), Strategies (limited perceived access to effective emotion regulation strategies), and Clarity (difficulty identifying and differentiating emotions). Items are rated on a five-point Likert scale, with higher scores indicating greater difficulty [1]. The French version has demonstrated a robust six-factor structure, excellent internal consistency (α = 0.92–0.94), and good temporal stability in French-speaking adult and university populations [47,48].

Reflective functioning, also referred to as mentalization, denotes the capacity to understand and interpret one’s own and others’ behaviors in terms of underlying mental states, such as emotions, beliefs, intentions, and desires. This capacity is generally regarded as an important component of socio-emotional development and may contribute to emotion regulation. Its impairment has been implicated in various forms of psychopathology, including BPD. Theoretically, mentalization is rooted in developmental models of attachment and affect regulation proposed by Fonagy and colleagues, according to which the capacity to represent mental states progressively develops through early interactions with attachment figures [27].

To assess this construct, participants completed the 54-item version of the Reflective Functioning Questionnaire (RFQ-54), originally developed by Fonagy and colleagues as a self-report measure of reflective functioning [35]. In the present study, only the eight items corresponding to the validated short version of the questionnaire (RFQ-8) were extracted and analyzed. This methodological choice was intended to rely on the version most widely used in recent studies and supported by the strongest psychometric evidence currently available. Although the RFQ-8 was not administered as an independent instrument, the eight items analyzed corresponded exactly to those comprising the validated short version and were extracted without any modification to their wording or response format.

Scoring was conducted in accordance with the original procedure described by Fonagy, including the specific recoding of items to derive the dimensions of certainty about mental states (RFQc) and uncertainty about mental states (RFQu). Items belonging to the RFQc subscale were recoded using the 3-2-1-0-0-0-0 scheme to reflect high levels of excessive certainty regarding mental states, historically interpreted as indicative of certain tendencies toward hypermentalizing, although this interpretation is currently subject to psychometric and conceptual debate. Conversely, items of the RFQu subscale were recoded according to the 0-0-0-0-1-2-3 scheme to capture elevated levels of uncertainty or opacity regarding mental states, corresponding to a form of hypomentalizing [35,37]. The RFQ-8 therefore allows for the assessment of two dimensions of reflective functioning corresponding to certainty and uncertainty about mental states. Whereas RFQu is generally interpreted as reflecting mentalizing difficulties consistent with hypomentalizing, the interpretation of RFQc as an indicator of hypermentalizing remains more controversial [11,36]. The psychometric properties of the French version were notably examined by Badoud et al., who confirmed a stable two-factor structure among French-speaking adolescents and young adults, with satisfactory internal consistency for both the RFQc and RFQu subscales [37].

Alexithymia was assessed using the French version of the Toronto Alexithymia Scale–20 items (TAS-20), a 20-item self-report questionnaire (5-point Likert scale) evaluating difficulties in identifying, describing, and processing emotions, with total scores from 20 to 100. The scale comprises three subscales: Difficulty Identifying Feelings (DIF), Difficulty Describing Feelings (DDF), and Externally Oriented Thinking (EOT, a concrete cognitive style with limited emotional introspection). The French version shows a factorial structure consistent with the original; good internal consistency for the total score, DIF, and DDF (EOT more moderate); and good temporal stability [49].

### 2.4. Statistical Analyses

All statistical analyses were performed using Stata software, version 14 (StataCorp, College Station, TX, USA). Participants were divided into two groups: a control group composed of individuals without a psychiatric history and a clinical group consisting of participants with borderline personality disorder.

Categorical variables were described using frequencies and percentages, whereas continuous variables were summarized using medians and interquartile ranges (P25–P75), given the non-normal distribution of several psychometric variables.

Group comparisons were conducted using the Chi-square test for categorical variables and the Wilcoxon–Mann–Whitney non-parametric test for continuous variables.

To examine differences associated with borderline status in relation to dimensions of mentalization, quantile regression models were applied to the RFQc and RFQu scores. Regression coefficients, presented together with their standard errors, correspond to median differences between participants with BPD and control subjects after adjustment for clinical and sociodemographic variables found to be significant in univariate analyses, namely: gender, personal psychiatric history, psychiatric comorbidities, substance use, family psychiatric history, history of suicide attempts, past and current psychological follow-up, psychotropic treatment, educational level, depressive symptomatology assessed using the BDI-II, and trait anxiety assessed using the STAI-T. Although several of these covariates are themselves closely associated with borderline personality disorder, their inclusion was intended to determine whether group differences in mentalization dimensions persisted beyond the broader clinical severity and treatment burden characteristic of BPD, rather than reflecting confounding by these correlated clinical features.

The relationships between mentalizing capacities and emotional dysregulation were explored using Spearman’s rank correlations between the RFQc/RFQu dimensions and DERS scores, both in the overall sample and within the BPD subgroup. Similar analyses were conducted to examine associations between dimensions of mentalization and the various components of impulsivity assessed by the UPPS-P.

Finally, multiple linear regression models were conducted to examine the associations between uncertainty regarding mental states (RFQu), generally interpreted as an indicator of mentalizing difficulties consistent with a form of hypomentalizing, and the emotional urgency dimensions of the UPPS-P (negative urgency and positive urgency), both in the overall sample and within the BPD subgroup. The models included alexithymia scores (TAS-20), depressive symptoms assessed using the BDI-II, and trait anxiety measured by the STAI-T as adjustment variables. In analyses conducted on the overall sample, the diagnosis of borderline personality disorder was also included as a covariate. For these models, bias-corrected bootstrap confidence intervals were computed for all regression coefficients to obtain more robust estimates of coefficient precision, particularly given the smaller sample size of the BPD subgroup (*n* = 45).

The threshold for statistical significance was set at *p* < 0.05 for all analyses. A Bonferroni correction was applied to correlational analyses involving multiple comparisons. Multicollinearity diagnostics (variance inflation factors, condition index) and overfitting diagnostics (training–test sample comparison) were computed for the quantile regression models comparing RFQc and RFQu scores between controls and individuals with BPD. For the multiple linear regression models predicting negative and positive urgency, multicollinearity was assessed using variance inflation factors, and overfitting was assessed using Leave-One-Out Cross-Validation (LOOCV), given that the smaller subgroup sample sizes precluded a training–test split. Bias-corrected bootstrap confidence intervals were computed for the multiple linear regression models predicting negative and positive urgency.

### 2.5. Ethical Considerations

This study was conducted in accordance with the ethical principles of the Declaration of Helsinki governing research involving human participants. The research protocol was approved by the Ethics Committee of the Faculty of Medicine of Erasme Hospital, Université libre de Bruxelles (Brussels, Belgium), under reference number P2020/111.

Prior to participation, all participants received detailed information regarding the objectives and procedures of the study. Written informed consent was obtained from all adult participants. For participants under the age of 18 years, written consent from at least one parent or legal guardian was also obtained in accordance with applicable ethical guidelines.

## 3. Results

### 3.1. Comparison of Clinical and Psychometric Characteristics Between Participants with BPD and Controls

The total sample comprised 166 participants, including 45 individuals with BPD and 121 non-clinical controls. Participants in the BPD group were significantly more likely to be female than control subjects (93.3% vs. 73.5%, *p* = 0.005). No significant differences were observed regarding age, student status, medical or surgical history, or alcohol consumption (Table 1).

Compared with the non-clinical group, participants with BPD reported significantly higher rates of personal psychiatric history, psychiatric comorbidities, illicit substance use, and family psychiatric history, as well as a greater number of previous suicide attempts and substances consumed (all comparisons: *p* < 0.001). Individuals with BPD were also more likely to have received past or current psychological treatment and to receive psychotropic medication (*p* < 0.001). Educational attainment was significantly lower in the BPD group (*p* = 0.035) (Table 1).

From a psychometric perspective, participants with BPD exhibited significantly higher RFQu scores than the non-clinical sample (11 [7–13] vs. 4 [2–7], *p* < 0.001), whereas no significant difference was observed for RFQc scores (*p* = 0.828) (Table 2).

The BPD group also displayed significantly higher levels of depressive symptoms (BDI-II), trait anxiety (STAI-T), global emotional dysregulation, and the majority of DERS dimensions (all comparisons: *p* < 0.001). Regarding impulsivity, participants with BPD obtained significantly higher scores on the negative urgency, positive urgency, lack of premeditation, and lack of perseverance dimensions of the UPPS-P (*p* < 0.001), whereas no significant difference was found for sensation seeking (Table 2).

Finally, participants with BPD reported significantly higher levels of total alexithymia (*p* = 0.022), primarily driven by the Difficulty Identifying Feelings (TAS-DIF) dimension (*p* < 0.001). In contrast, Externally Oriented Thinking (TAS-EOT) scores were slightly lower in the BPD group (*p* = 0.014), whereas no significant difference was found for the Difficulty Describing Feelings (TAS-DDF) dimension (*p* = 0.072) (Table 2).

### 3.2. Adjusted Analyses of Mentalization Dimensions According to Borderline Status (Table 3)

After adjustment for gender, personal psychiatric history, psychiatric comorbidities, substance use, family psychiatric history, history of suicide attempts, past and current psychological follow-up, psychotropic treatment, educational level, BDI-II depression scores, and STAI-T trait anxiety scores, participants with borderline personality disorder continued to exhibit significantly higher levels of uncertainty regarding mental states (RFQu) than non-clinical controls (β = 4.7; SE = 1.7; *p* < 0.05).

In contrast, no significant difference was observed for RFQc scores after adjustment (β = 2.0; SE = 1.7; *p* > 0.05).

Overall, after accounting for the covariates examined, the differences observed between participants with BPD and controls primarily concerned the uncertainty regarding the mental states (RFQu) dimension, whereas no significant difference was found for RFQc.

**Table 3:** Adjusted results for mentalization parameters (*n* = 166).

Variables	b_a1_ (ES) Controls vs. BPD Individuals	95% CI	*p*-Value	Pseudo-R^2^
RFQc	2.0 (1.7)	−1.32 to 5.32	0.236	0.082
RFQu	4.7 (1.7)	1.31 to 8.03	0.007	0.305

b_a1_ (ES): quantile regression coefficient adjusted (standard error). These coefficients are the difference in median between Controls and BPD individuals, adjusted for gender, psychiatric history, psychiatric comorbidities, drugs, family psychiatric history, history of suicide attempts, past psychological follow-up, actual psychological follow-up, psychotropic treatment, educational level, BDI-II scores and STAI-T scores.

### 3.3. Associations Between Mentalization and Emotional Dysregulation in the Overall Sample (Table 4)

In the total sample, Spearman correlations were conducted to examine the associations between the mentalization dimensions assessed by the RFQ-8 and the various dimensions of emotional dysregulation measured by the DERS.

RFQu scores were positively and significantly correlated with all dimensions of emotional dysregulation after Bonferroni correction. The strongest associations involved emotional clarity difficulties (DERS Clarity; r = 0.676), impulse control difficulties (DERS Impulse; r = 0.634), limited access to emotion regulation strategies (DERS Strategies; r = 0.611), and the total DERS score (r = 0.728) (all *p* < 0.05 after correction). Significant correlations were also observed with Non-Acceptance (DERS Nonacceptance; r = 0.454), Goals (r = 0.453), and Awareness (r = 0.475).

Conversely, RFQc scores were negatively correlated with certain DERS dimensions. Significant associations after Bonferroni correction were observed for Impulse (r = −0.391), Clarity (r = −0.267), and the total DERS score (r = −0.284).

Overall, these findings indicate that greater uncertainty regarding mental states is strongly associated with more severe global emotional dysregulation, particularly in domains involving the understanding of emotions and behavioral control under emotional distress.

**Table 4:** Correlation between DERS dimensions and mentalization parameters for whole sample (*n* = 166).

	RFQc	RFQu
DERS Non-Acceptance	−0.097	0.454 *
DERS Goals	−0.152	0.453 *
DERS Impulse	−0.391 *	0.634 *
DERS Awareness	−0.147	0.475 *
DERS Clarity	−0.267 *	0.676 *
DERS Strategies	−0.195	0.611 *
DERS total	−0.284 *	0.728 *

* *p* < 0.05 after Bonferroni correction; correlations with an absolute value ≥ 0.40 (|r| ≥ 0.40), whether positive or negative, are considered of moderate to large magnitude and are highlighted to facilitate clinical interpretation. Bold formatting is used to highlight correlations with an absolute value ≥ 0.40 (|r| ≥ 0.40), whether positive or negative, as these are considered of moderate to large magnitude and are emphasized to facilitate clinical interpretation.

### 3.4. Associations Between Mentalization and Emotional Dysregulation Among Participants with BPD (Table 5)

Within the BPD group, Spearman correlations were conducted between the RFQ-8 dimensions and the various DERS dimensions.

RFQu scores were positively associated with all DERS dimensions. After Bonferroni correction, the associations remained significant for impulse control difficulties (DERS Impulse; r = 0.608) and the total DERS score (r = 0.634; *p* < 0.05 after correction).

Positive correlations of moderate magnitude were also observed between RFQu and the Goals, Awareness, Clarity, and Strategies dimensions, although these associations no longer reached significance after correction for multiple comparisons. No significant association was found between RFQu and the Non-Acceptance dimension.

Regarding RFQc, no significant correlations were observed with any DERS subscale or with the total emotional dysregulation score.

Overall, the strongest associations involved impulse control difficulties and the total emotional dysregulation score, whereas no significant association was observed for RFQc. These findings suggest that, among young adults with BPD, uncertainty regarding mental states is primarily associated with global difficulties in emotion regulation and, more specifically, with difficulties controlling impulses in the context of emotional distress.

**Table 5:** Correlation between DERS dimensions and mentalization parameters for BPD subjects (*n* = 45).

	RFQc	RFQu
DERS Non-Acceptance	−0.084	0.272
DERS Goals	0.131	0.314
DERS Impulse	0.244	0.608 *
DERS Awareness	0.131	0.342
DERS Clarity	0.161	0.356
DERS Strategies	−0.103	0.441
DERS total	0.090	0.634 *

* *p* < 0.05 after Bonferroni correction; correlations with an absolute value ≥ 0.40 (|r| ≥ 0.40), whether positive or negative, are considered of moderate to large magnitude and are highlighted to facilitate clinical interpretation. Bold formatting is used to highlight correlations with an absolute value ≥ 0.40 (|r| ≥ 0.40), whether positive or negative, as these are considered of moderate to large magnitude and are emphasized to facilitate clinical interpretation.

### 3.5. Associations Between Mentalization and Impulsivity in the Overall Sample (Table 6)

Spearman correlations were conducted in the overall sample to examine associations between the mentalization dimensions assessed by the RFQ-8 and the various dimensions of impulsivity measured by the UPPS-P.

RFQu scores were positively and significantly correlated with several dimensions of impulsivity after Bonferroni correction. The strongest associations involved negative urgency (r = 0.628), followed by lack of premeditation (r = 0.461), positive urgency (r = 0.389), and lack of perseverance (r = 0.357) (all *p* < 0.05 after correction). In contrast, no significant association was observed between RFQu and sensation seeking (r = 0.083).

Regarding RFQc, significant negative correlations were found with negative urgency (r = −0.519) and positive urgency (r = −0.246), suggesting that greater certainty regarding mental states was associated with lower levels of emotion-related impulsivity. A negative correlation was also observed with lack of premeditation (r = −0.214), although it did not remain significant after Bonferroni correction. No significant associations were found with lack of perseverance or sensation seeking.

Overall, the strongest associations involved the emotional urgency dimensions, particularly negative urgency, whereas no significant association was observed between RFQu and sensation seeking. These findings suggest that the relationships between uncertainty regarding mental states and impulsivity primarily concern the emotional dimensions of the UPPS-P.

**Table 6:** Correlation between UPPS dimensions and mentalization parameters for whole sample (*n* = 166).

	RFQc	RFQu
UPPS urgencies negative	−0.519 *	0.628 *
UPPS urgencies positive	−0.246 *	0.389 *
UPPS lacks premeditation	−0.214	0.461 *
UPPS lacks perseverance	−0.073	0.357 *
UPPS seeks sensation	−0.007	0.083

* *p* < 0.05 after Bonferroni correction; correlations with an absolute value ≥ 0.40 (|r| ≥ 0.40), whether positive or negative, are considered of moderate to large magnitude and are highlighted to facilitate clinical interpretation. Bold formatting is used to highlight correlations with an absolute value ≥ 0.40 (|r| ≥ 0.40), whether positive or negative, as these are considered of moderate to large magnitude and are emphasized to facilitate clinical interpretation.

### 3.6. Associations Between Mentalization and Impulsivity Among Participants with BPD (Table 7)

Within the BPD group, Spearman correlations were conducted between the RFQ-8 dimensions and the various UPPS-P impulsivity dimensions.

RFQu scores were positively associated with all impulsivity dimensions, with particularly marked correlations for negative urgency (r = 0.432) and positive urgency (r = 0.418). After Bonferroni correction, only the association between RFQu and negative urgency remained statistically significant (*p* < 0.05).

Positive correlations of more moderate magnitude were also observed between RFQu and lack of premeditation (r = 0.347) and lack of perseverance (r = 0.204), whereas no association was found with sensation seeking (r = −0.030).

Regarding RFQc, no significant correlations were observed with any of the UPPS-P dimensions. The coefficients obtained were small and non-significant, including those for the emotional dimensions of impulsivity.

Overall, the strongest associations involved the emotional urgency dimensions, particularly negative urgency, whereas no association was observed with sensation seeking or RFQc.

**Table 7:** Correlation between UPPS dimensions and mentalization parameters for BPD subjects (*n* = 45).

	RFQc	RFQu
UPPS urgencies negative	−0.230	0.432 *
UPPS urgencies positive	0.048	0.418
UPPS lacks premeditation	0.024	0.347
UPPS lacks perseverance	−0.005	0.204
UPPS seeks sensation	0.131	−0.030

* *p* < 0.05 after Bonferroni correction; correlations with an absolute value ≥ 0.40 (|r| ≥ 0.40), whether positive or negative, are considered of moderate to large magnitude and are highlighted to facilitate clinical interpretation. Bold formatting is used to highlight correlations with an absolute value ≥ 0.40 (|r| ≥ 0.40), whether positive or negative, as these are considered of moderate to large magnitude and are emphasized to facilitate clinical interpretation.

### 3.7. Association Between Uncertainty Regarding Mental States and Negative Urgency After Adjustment for Alexithymia and Clinical Variables

Multiple linear regression models were conducted to examine whether RFQu remained associated with negative urgency independently of alexithymia, anxiety–depressive symptomatology, and borderline status.

In the overall sample, RFQu scores were significantly associated with UPPS-P negative urgency after adjustment for TAS-20 scores, depressive symptoms (BDI-II), trait anxiety (STAI-T), and borderline diagnosis (β = 0.42; bootstrap SE = 0.07; *p* < 0.001; bias-corrected 95% CI [0.30, 0.56]). The model explained approximately 40% of the variance in negative urgency (R^2^ = 0.3995) (Table 8).

In contrast, neither total alexithymia (TAS-20), depression scores, trait anxiety, nor borderline diagnosis remained significantly associated with negative urgency in this model.

Similar analyses conducted within the BPD subgroup confirmed these findings. After adjustment for TAS-20 scores, depression, and trait anxiety, RFQu scores remained significantly associated with negative urgency (β = 0.41; bootstrap SE = 0.10; *p* < 0.001; bias-corrected 95% CI [0.21, 0.59]), whereas alexithymia and anxiety–depressive variables were not significantly associated with the model. The model explained approximately 35% of the variance in negative urgency within the BPD group (R^2^ = 0.3540) (Table 9).

### 3.8. Exploratory Associations Between RFQu and Positive Urgency

Multiple linear regression models were also conducted to examine the associations between RFQu and positive urgency of the UPPS-P after adjustment for alexithymia, depressive symptoms, trait anxiety, and borderline status.

In the overall sample, after adjustment for TAS-20 scores, depressive symptoms (BDI-II), trait anxiety (STAI-T), and borderline diagnosis, RFQu was no longer significantly associated with positive urgency when bias-corrected bootstrap confidence intervals were used (β = 0.12; bootstrap SE = 0.08; *p* = 0.104; bias-corrected 95% CI [−0.02, 0.27]). None of the other covariates included in the model were significantly associated with positive urgency. The model explained approximately 16% of the variance in positive urgency (R^2^ = 0.1633) (Table 10).

Within the BPD subgroup, the association between RFQu and positive urgency, after adjustment for alexithymia, depression, and trait anxiety, did not reach statistical significance when bias-corrected bootstrap confidence intervals were used (β = 0.21; bootstrap SE = 0.12; *p* = 0.087; bias-corrected 95% CI [−0.04, 0.44]). None of the other variables included in the model were significantly associated with positive urgency. The model explained approximately 13% of the variance in this impulsivity dimension (R^2^ = 0.1329) (Table 11).

These findings do not provide statistical support for an association between uncertainty regarding mental states and impulsive tendencies occurring in positively activated emotional contexts, once adjusted models are evaluated using bias-corrected bootstrap confidence intervals. The point estimates were comparable in direction and magnitude to those observed for negative urgency, but with considerably greater imprecision, and should therefore not be interpreted as evidence of an association.

## 4. Discussion

### 4.1. Main Findings

The present study aimed to examine the relationships between mentalizing difficulties, emotional dysregulation, and emotion-related impulsivity among young adults with BPD. More specifically, it pursued three main objectives: to determine whether mentalizing difficulties were more pronounced among participants with BPD than among non-clinical controls, to explore the associations among mentalization, emotional dysregulation, and impulsivity, and to evaluate the specific contribution of mentalization to emotion-related impulsivity beyond alexithymia and anxiety–depressive distress.

Overall, the findings largely support the hypotheses formulated. Participants with BPD reported significantly higher levels of uncertainty regarding mental states than controls, and this difference remained significant after adjustment for the principal clinical and sociodemographic factors. Uncertainty regarding mental states was also associated with greater emotional dysregulation and with the emotional dimensions of impulsivity, particularly negative urgency. Finally, multivariate analyses showed that uncertainty regarding mental states remained associated with the emotional urgency dimensions after adjustment for alexithymia, anxiety and depressive symptomatology, and, in the overall sample, borderline status. These observations suggest that mentalizing difficulties may be specifically associated with the emotion-related impulsivity observed in BPD, beyond difficulties in identifying and verbalizing affective experiences.

### 4.2. Mentalizing Difficulties and Borderline Status in Young Adults

Participants with BPD exhibited a clinical profile consistent with the existing literature, characterized by higher levels of depressive and anxiety symptoms, as well as increased emotion-related impulsivity. These findings are in line with contemporary models of BPD, which describe the disorder as being marked by substantial affective instability, heightened emotional reactivity, and a tendency toward impulsive behaviors in contexts of emotional distress [7]. Furthermore, the elevated rates of anxiety and depressive symptomatology observed in our sample are consistent with numerous studies highlighting a high prevalence of mood and anxiety disorder comorbidities among individuals with BPD [50,51]. They are also congruent with recent studies conducted among young adults with BPD emphasizing the central role of emotional dysregulation and emotion-related impulsivity in this population [3,9]. This severity is further contextualized by a recent large-scale meta-analysis showing that, among 12 psychiatric disorders, BPD exhibited the largest case–control effect size for DERS-assessed emotion dysregulation (Hedges’ g = 2.55), significantly greater than that observed for most other diagnoses, while confirming that emotion dysregulation constitutes a transdiagnostic feature present with large effect sizes across the disorders examined [52].

Among the observed differences, the marked increase in RFQu scores among participants with BPD appears particularly noteworthy, as it suggests the presence of mentalizing difficulties extending beyond anxiety–depressive symptomatology alone. This interpretation is reinforced by the adjusted analyses demonstrating that, after controlling for all covariates, the median RFQu score remained 4.7 points higher among participants with BPD compared with controls. The magnitude of this residual difference suggests that difficulties in representing mental states cannot be reduced to an expression of general affective distress or a greater overall psychopathological burden. Rather, these difficulties may constitute a relatively specific dimension of borderline functioning.

The robustness of the association between borderline diagnosis and elevated RFQu scores after adjustment is consistent with several studies demonstrating that hypomentalizing constitutes a particularly salient characteristic of BPD. Perroud and colleagues observed that adults with BPD reported significantly higher levels of uncertainty regarding mental states compared with both healthy controls and patients with ADHD, even after adjustment for depressive symptomatology and mindfulness capacities [43]. Similarly, Badoud and colleagues showed that RFQu scores mediated the relationship between insecure attachment and borderline diagnostic status, underscoring the central role of hypomentalizing in the psychopathology of the disorder [53]. These findings converge with those of Morandotti and colleagues, who demonstrated that RFQu significantly discriminated patients with BPD from healthy controls and independently predicted clinician-rated disorder severity after controlling depressive symptoms [54]. In a meta-analysis encompassing 34 studies, Bora further confirmed that impairments in self-reported reflective functioning represented the most robust characteristic among measures of social cognition in BPD, with a particularly large effect size [55]. More recently, Kurt and colleagues showed that hypomentalizing was among the variables that most effectively discriminated patients with BPD from comparison groups, alongside epistemic mistrust and attachment anxiety [56].

Although our study does not allow for direct testing of these developmental mechanisms, the persistence of RFQu differences after adjustment appears consistent with the models proposed by Fonagy and Luyten, who suggested that the mentalizing difficulties observed in BPD do not merely reflect heightened emotional reactivity but rather correspond to a more fundamental developmental vulnerability in the capacity to represent one’s own and others’ mental states [12]. According to this model, insecure attachment experiences and early invalidating environments compromise the development of reflective functioning, rendering individuals particularly vulnerable to losing their mentalizing capacities in contexts characterized by heightened emotional and interpersonal activation [12,32]. From a neurobiological perspective, Fonagy, Luyten and Strathearn proposed a developmental “switch model” according to which, beyond a certain threshold of attachment-system activation, controlled mentalization becomes deactivated in favor of automatic and prementalizing modes of processing [57]. Within this framework, individuals with BPD would be characterized by a particularly low threshold for such deactivation, making them especially vulnerable to losses of mentalization in emotionally charged interpersonal contexts [57].

This interpretation is also consistent with the work of Quek and colleagues, who demonstrated that reflective functioning partially mediated the relationship between childhood emotional maltreatment and borderline pathology during adolescence [42], suggesting that early adversities may contribute to the later development of the mentalizing difficulties observed in BPD.

Taken together, our findings appear more compatible with models emphasizing mentalizing difficulties associated with uncertainty regarding mental states than with a predominant role for hypermentalization phenomena assessed through RFQc in young adults with BPD. Hypomentalizing, characterized by marked uncertainty concerning one’s own and others’ mental states and by difficulties constructing coherent representations of interpersonal behaviors, has been described by several authors as the dysfunctional mode of mentalization most directly involved in the clinical manifestations of the disorder [12,32,53]. This perspective is consistent with the work of Asztalos and colleagues, who demonstrated that certain ineffective mentalizing modes, particularly hypomentalizing, occupied a central position within the network of difficulties associated with BPD, suggesting that the relationships between mentalization and borderline psychopathology are specific rather than diffuse [58].

By contrast, the RFQc dimension did not remain significantly associated with borderline status after adjustment (β = 2.0; SE = 1.7; *p* > 0.05). This absence of a significant association should be interpreted cautiously and does not permit the conclusion that hypermentalization is absent in BPD. Several authors have highlighted the psychometric limitations of RFQc as an indicator of hypermentalization [11,36]. High certainty regarding mental states may reflect adaptive as well as dysfunctional processes, thereby limiting the discriminant validity of this subscale. Studies employing behavioral measures, notably the Movie for the Assessment of Social Cognition (MASC), have consistently identified more frequent hypermentalizing errors among adolescents and adults with BPD or elevated borderline traits [33,57,58,59,60]. The meta-analysis conducted by Németh and colleagues further suggests that social cognitive difficulties in BPD primarily concern reasoning about mental states rather than simple perceptual decoding of those states [34]. Consequently, the absence of a significant RFQc association in our study most likely reflects limitations of the instrument rather than the true absence of hypermentalizing phenomena among participants with BPD.

Finally, the study by Lopez-Villatoro and colleagues highlights the heterogeneity of mentalization profiles in BPD according to associated comorbidities. The authors showed that patients presenting with comorbid suicidal behavior exhibited a greater number of undermentalizing errors on the MASC, whereas other clinical profiles appeared to be more closely associated with forms of hypermentalization or non-mentalization [61]. Such heterogeneity may contribute to explaining the discrepancies observed in the literature regarding the predominant type of mentalizing dysfunction. In our cohort of young adults, characterized by the high prevalence of suicidal behaviors and depressive symptomatology, the findings observed in the present study appear compatible with this hypothesis of heterogeneous mentalization profiles in BPD.

Overall, the findings of this study suggest that uncertainty regarding mental states, generally interpreted as reflecting mentalizing difficulties consistent with a form of hypomentalizing, remains associated with borderline status after accounting for anxiety–depressive distress and the other clinical factors considered. These observations reinforce the notion that the mentalizing difficulties observed among young adults with BPD cannot be reduced to the expression of general psychopathology or to the severity of anxiety and depressive symptoms alone but may instead be specifically associated with the clinical manifestations of BPD.

Although these findings do not allow for conclusions regarding the specificity of BPD in the absence of a psychiatric comparison group—a limitation reinforced by recent evidence that emotion dysregulation is transdiagnostic in nature [52]—they nevertheless appear consistent with developmental models of mentalization and with empirical evidence suggesting that difficulties in representing mental states constitute a particularly salient characteristic of borderline functioning.

### 4.3. Mentalizing Difficulties and Emotional Dysregulation in BPD

States is associated with greater difficulties in emotion regulation, which appear to constitute important mechanisms in the development and maintenance of various forms of psychopathology [40,61,62,63,64]. From this perspective, the emotional difficulties observed in individuals with compromised mentalization are not independent of their capacities to represent mental states but are instead closely intertwined with them.

The particularly strong association observed with the Clarity dimension is especially noteworthy. This dimension refers to the ability to identify, differentiate, and understand one’s own emotions. The work of Kahya and Munguldar (2023) specifically demonstrates that uncertainty regarding mental states is associated with lower emotional clarity and greater difficulties in emotion regulation [40]. More broadly, several authors have emphasized that mentalization facilitates the understanding of internal experiences and contributes to making affective states more intelligible and coherent [62,65]. Within this framework, when mental states are experienced with uncertainty, emotions may become more difficult to identify, differentiate, and interpret, fostering an emotional experience characterized by confusion and a lack of clarity. The fact that the strongest correlation observed after the total DERS score involved precisely the Clarity dimension appears consistent with this hypothesis.

Another noteworthy finding concerns the Awareness dimension of the DERS. Although the association between RFQu and reduced emotional awareness did not remain significant after correction for multiple comparisons within the BPD subgroup, a positive correlation was observed in the overall sample. This finding deserves attention considering our previous work, in which reduced emotional awareness emerged as one of the dimensions of emotional dysregulation most strongly associated with BPD among young adults [3]. Emotional awareness and mentalization indeed share a common focus on attention directed toward internal experiences, although they do not refer to identical processes. Emotional awareness primarily concerns the capacity to recognize and attend to one’s affective states, whereas mentalization involves a more complex elaboration aimed at attributing meaning to one’s own and others’ mental states and integrating them into the understanding of behavior [12,32]. This articulation may also be related to the concept of Wise Mind proposed by Linehan, which refers to the capacity to integrate emotional and rational information to achieve a more balanced understanding of internal experience. Although these constructs are not interchangeable, they converge on the notion that greater attention to internal experiences promotes more adaptive emotion regulation [8].

An important question, however, was whether these associations would persist within the BPD subgroup, characterized by globally elevated levels of emotional dysregulation. Within this subgroup, the associations between RFQu and the DERS remained significant after correction for multiple comparisons only for the total emotional dysregulation score (r = 0.634) and the Impulse dimension (r = 0.608). Correlations with the remaining dimensions, although of moderate magnitude, no longer survived statistical correction.

The strong correlations observed with the Impulse dimension suggest that mentalizing difficulties are associated with a reduced capacity to maintain behavioral control as emotional intensity increases. This interpretation is consistent with studies conducted in the field of borderline personality disorder, where mentalizing difficulties are closely linked to emotional dysregulation and impulsivity. Euler et al., for example, demonstrated that the interpersonal difficulties observed in patients with BPD were associated with disturbances in mentalization, emotional dysregulation, and impulsivity [41]. Similarly, Sharp et al. showed that difficulties in emotion regulation mediated the relationship between disturbances in mentalization and borderline traits in adolescents [33]. Although the latter study primarily focused on overmentalizing assessed using the Movie for the Assessment of Social Cognition (MASC), it nevertheless supports the notion that mentalizing difficulties and emotional dysregulation are tightly interwoven within borderline functioning. In the present study, the persistence of a significant association between RFQu and the Impulse dimension within the BPD subgroup suggests that difficulties in behavioral control under emotional distress may represent a particularly salient manifestation of mentalizing difficulties in this population.

The absence of significant associations between RFQu and certain DERS dimensions, particularly Clarity and Awareness, after correction within the BPD subgroup should nevertheless be interpreted cautiously. The relatively small size of this subgroup limits the statistical power available to detect certain associations, and a restriction of variance is also possible in a population characterized by globally elevated levels of emotional dysregulation. Consequently, these findings do not permit the conclusion that no association exists between mentalizing difficulties and these emotional dimensions among participants with BPD.

In contrast, the associations involving the RFQc dimension appeared more limited. In the overall sample, RFQc was correlated with the total DERS score as well as with the Clarity and Impulse dimensions, although the coefficients were substantially smaller than those observed for RFQu. Within the BPD subgroup, no correlations between RFQc and the DERS dimensions remained significant after correction for multiple comparisons. These findings suggest that the emotional difficulties observed in our sample are more closely related to uncertainty regarding mental states than to elevated levels of certainty. They are therefore consistent with studies showing that the RFQu dimension generally constitutes the indicator most strongly associated with psychopathological manifestations and difficulties in emotion regulation [40,41].

Taken together, these findings suggest that, among young adults with BPD, mentalizing difficulties are primarily associated with global difficulties in emotion regulation and, more specifically, with difficulties maintaining behavioral control under emotional distress. The persistence of the association between RFQu and the Impulse dimension within the BPD subgroup underscores the robustness of the link between uncertainty regarding mental states and difficulties maintaining behavioral control as emotional intensity increases.

This observation appears particularly relevant considering the analyses examining emotion-related impulsivity, which allow for investigation of whether these difficulties in regulation under emotional distress also extend to dispositional tendencies to act impulsively in response to intense affective states.

### 4.4. Uncertainty Regarding Mental States: Preferential Associations with the Emotional Urgency Dimensions

The findings of the present study indicate that mentalizing difficulties, and more specifically uncertainty regarding mental states (RFQu), are primarily associated with the emotional dimensions of impulsivity. In the overall sample, RFQu scores were positively correlated with several UPPS-P dimensions, with particularly strong associations observed for negative urgency, followed by positive urgency. More modest correlations were also found with lack of premeditation and lack of perseverance, whereas no association emerged with sensation seeking. This pattern suggests that mentalizing difficulties are not associated with a global and undifferentiated form of impulsivity but rather with dimensions involving difficulties in regulating or controlling behavior, particularly when emotions are strongly activated.

The associations observed lack of premeditation and lack of perseverance in the overall sample also warrant consideration. They may suggest that a reduced capacity to represent and understand mental states limits, to some extent, the anticipation of the future consequences of one’s behavior or the ability to maintain goal-directed behavior when internal experiences become confusing or overwhelming. However, the more modest magnitude of these associations, together with their failure to persist within the BPD subgroup after correction for multiple comparisons, suggests that mentalizing difficulties are more closely linked to the emotional dimensions of impulsivity than to the other facets of the UPPS-P model.

This observation is consistent with contemporary models of mentalization, according to which reflective capacities become particularly vulnerable as emotional intensity increases. Fonagy et al. (2011) proposed that activation of the attachment system and emotional distress temporarily reduce mentalizing capacities, favoring more automatic and less reflective modes of processing [57]. Within this framework, impulsive behaviors may be understood as attempts to respond rapidly to emotional experiences that have not been sufficiently elaborated through mentalization, rather than as the expression of a simple generalized tendency toward risk-taking [57].

The articulation between these findings and those reported in the previous section appears particularly noteworthy. We demonstrated that, among young adults with BPD, uncertainty regarding mental states remained associated with difficulties in maintaining behavioral control under emotional distress, as assessed by the Impulse dimension of the DERS. By contrast, negative urgency within the UPPS-P refers to a more dispositional tendency to act impulsively under the influence of intense negative emotions. The fact that RFQu was associated with both dimensions suggests that mentalizing difficulties may be involved both in immediate difficulties controlling behavior in the face of emotional distress and in more stable dispositions to respond impulsively to negative affect.

The association observed with negative urgency appears particularly relevant in the context of BPD. This dimension refers to the tendency to act impulsively when confronted with intense negative emotions. Several studies suggest that mentalizing difficulties limit the ability to identify, understand, and regulate internal states, thereby increasing the likelihood of immediate behavioral responses to emotional distress [40,41]. Extending these observations, Kurt et al. argued that hypomentalizing compromises the understanding of mental states and promotes the use of impulsive or self-damaging behaviors as concrete and immediate responses to psychological suffering. The authors proposed that, when the mental elaboration of emotional experience becomes insufficient, action may substitute for reflection to rapidly reduce distress [56]. This conceptualization closely resembles the notion of negative urgency and provides a compelling explanatory framework for the present findings.

The associations observed with positive urgency in the overall sample provide complementary insights. They suggest that mentalizing difficulties are not exclusively linked to impulsive behaviors triggered by negative emotions but, more broadly, to situations characterized by heightened affective activation. A reduced capacity to mentalize may limit the maintenance of a reflective distance from intense emotional states, thereby increasing the likelihood of impulsive behaviors in response to strongly activated affects, regardless of their emotional valence. Nevertheless, the fact that only negative urgency remained associated with RFQu within the BPD subgroup after correction for multiple comparisons suggests that negative emotions occupy a particularly central role in this population. This observation is consistent with contemporary models of BPD, which conceptualize impulsive behaviors as being closely tied to attempts to regulate intense negative affect.

The absence of an association between RFQu and sensation seeking is also particularly informative. It suggests that mentalizing difficulties are not related to a general propensity to seek novel, stimulating, or potentially risky experiences but rather to forms of impulsivity involving heightened reactivity to emotional states. This specificity strengthens the hypothesis that mentalizing difficulties are more strongly implicated in the emotional forms of impulsivity than in a generalized impulsive disposition.

The findings observed for the RFQc dimension should likewise be interpreted with caution. In the overall sample, greater certainty regarding mental states was associated with lower levels of both negative urgency and positive urgency. However, these associations were weaker than those observed for RFQu and were no longer evident within the BPD subgroup after correction for multiple comparisons. Given the conceptual and psychometric limitations discussed regarding RFQc as an indicator of hypermentalizing [11,36], these findings should be considered exploratory. Nevertheless, they suggest that the emotional and behavioral difficulties observed in our sample are more closely related to uncertainty regarding mental states than to elevated levels of certainty about them. This observation is consistent with studies identifying RFQu as the dimension of mentalization most strongly associated with emotional and behavioral difficulties in BPD [40,41,56].

Finally, the persistence of the association between RFQu and negative urgency after correction for multiple comparisons within the BPD subgroup probably constitutes the most salient finding of this series of analyses. Taken together, these results suggest that mentalizing difficulties are more strongly associated with forms of impulsivity involving reactivity to emotional states than with a general propensity toward sensation seeking. This specificity appears particularly pronounced among young adults with BPD, in whom the tendency to act impulsively under the influence of intense negative emotions seems to be closely linked to uncertainty regarding mental states.

Importantly, the absence of significant associations for some UPPS-P dimensions within the BPD subgroup should be interpreted cautiously. Given the relatively small size of this subgroup and the possibility of restricted variability in a clinically severe sample, smaller associations may have remained undetected.

### 4.5. Mentalizing Difficulties and Emotion-Related Impulsivity: A Specific Association Beyond Alexithymia

One of the most striking findings of the present study is that uncertainty regarding mental states remained associated with negative urgency independently of alexithymia, anxiety–depressive symptomatology, and, in the overall sample, borderline diagnosis. This association also persisted within the BPD subgroup itself. More specifically, RFQu remained significantly associated with negative urgency both in the overall sample and in the BPD group, whereas none of the other variables included in the models retained an independent association. In contrast, the corresponding associations with positive urgency did not reach statistical significance once bias-corrected bootstrap confidence intervals were used, both in the overall sample and within the BPD subgroup, despite point estimates of comparable direction and magnitude. This asymmetry was further corroborated by model diagnostics: Leave-One-Out Cross-Validation indicated no substantial overfitting for the negative urgency model within the BPD subgroup (adjusted R^2^ decrease of 18.25%), whereas the corresponding positive urgency model showed a marked decrease in cross-validated performance (adjusted R^2^ decrease of 65.24%), indicating that its already non-significant estimates should be interpreted with particular caution. This pattern suggests that the independent association between uncertainty regarding mental states and emotion-related impulsivity, beyond alexithymia and anxiety–depressive distress, is more robustly established for negative urgency than for positive urgency.

These findings extend those discussed in the preceding sections. We demonstrated that uncertainty regarding mental states was associated both with difficulties in maintaining behavioral control under emotional distress, as assessed by the Impulse dimension of the DERS, and with the emotional dimensions of impulsivity measured by the UPPS-P. Whereas the Impulse dimension reflects difficulties maintaining behavioral control as emotional intensity increases, negative urgency refers to a more stable dispositional tendency to act impulsively under the influence of intense negative affect. The fact that RFQu remained associated with both the DERS Impulse dimension and negative urgency strengthens the hypothesis that mentalizing difficulties may be involved both in immediate difficulties in behavioral regulation under distress and in more enduring tendencies to act impulsively in response to intense negative emotions.

These findings also differ from those observed in our previous study focusing on alexithymia. In that study, although alexithymia was significantly higher among young adults with BPD, the associations between alexithymia dimensions and emotion-related impulsivity observed in the overall sample were attenuated and no longer reached statistical significance within the BPD subgroup. Given the modest size and clinical severity of this subgroup, these findings should be interpreted cautiously [25]. In contrast, in the present study, uncertainty regarding mental states remained associated with negative urgency within the BPD group itself, even after adjustment for alexithymia, depressive symptoms, and trait anxiety. The corresponding association with positive urgency did not reach statistical significance in this study.

Part of this observation may be attributable to the conceptual overlap between these two constructs. Certain dimensions of alexithymia, particularly difficulties identifying and understanding internal states, share theoretical similarities with mentalizing difficulties associated with uncertainty regarding mental states. It is therefore possible that RFQu captures part of the variance shared between these constructs, which could account for why alexithymia no longer remained significantly associated with the emotional urgency dimensions after adjustment. This interpretation is consistent with studies demonstrating that difficulties identifying emotions are associated with negative urgency and certain forms of emotion-related impulsivity [24], as well as with research highlighting the relationships among alexithymia, emotional dysregulation, and impulsivity [22].

However, the present findings also suggest a qualitative distinction between alexithymia and mentalization. Alexithymia, as assessed by the TAS-20, primarily refers to difficulties identifying and describing emotions, as well as a tendency toward externally oriented thinking [66]. Mentalization, by contrast, refers to the capacity to understand one’s own and others’ behaviors considering underlying mental states, such as emotions, desires, beliefs, and intentions, and to use these representations to make sense of subjective experience and guide behavior [27]. In other words, alexithymia primarily addresses the question, “What am I feeling?” whereas mentalization involves the broader capacity to ask, “What does what I am feeling mean? Why am I feeling this? What is happening in my mind and in the minds of others?” This conceptual distinction may help account for why RFQu retained an independent association with the emotional urgency dimensions, whereas TAS-20 scores lost significance in the multivariate models.

Thus, tendencies to act impulsively under the influence of intense emotions may involve difficulties in representing and interpreting mental states that extend beyond the contribution of alexithymic difficulties as assessed in the present study. The present findings are therefore consistent with the hypothesis that mentalizing difficulties may be particularly relevant to forms of emotion-related impulsivity characterized by acting under emotional distress, especially negative urgency. Taken together, these findings suggest that uncertainty regarding mental states may account for additional variance in emotion-related impulsivity beyond the contribution of alexithymic difficulties as assessed in the present study. Rather than indicating that alexithymia is unrelated to impulsive manifestations in BPD, these observations suggest that alexithymia and mentalization may capture partially overlapping yet distinct aspects of emotional functioning, each being differently associated with the expression of emotion-related impulsivity. Nevertheless, given the cross-sectional nature of this study, these results do not permit causal inferences or support the conclusion that mentalization necessarily occupies a more proximal position within the psychopathological pathway.

This interpretation is consistent with mentalization-based models, according to which the capacity to maintain sufficiently stable and nuanced representations of mental states constitutes an important prerequisite for the regulation of intense affective experiences [12,27]. When this capacity is compromised, individuals may become more inclined to respond to emotional states through immediate behaviors aimed at reducing internal tension rather than elaborating on their psychological experience. Several empirical studies have also demonstrated associations between hypomentalizing, emotional dysregulation, and core manifestations of borderline personality disorder, including impulsivity [40,53,67].

Furthermore, the persistence of the associations between RFQu and negative urgency within the BPD subgroup appears particularly noteworthy. Despite relative diagnostic homogeneity, individual differences in mentalizing capacities continued to account for a significant proportion of the variance in negative urgency. This observation suggests that assessing mentalization in patients with BPD does not merely provide information redundant with the diagnosis itself. On the contrary, it may contribute to a more refined characterization of the clinical heterogeneity observed within this population and help identify patients who are particularly prone to acting impulsively under the influence of intense negative emotions.

These findings are especially meaningful in light of the developmental context of our sample, which consists of young adults aged 16 to 25 years. The mentalizing difficulties observed in these patients may reflect developmental vulnerabilities that progressively affect the capacity to represent mental states in situations of interpersonal stress. According to the model proposed by Fonagy and colleagues, early disturbances in attachment relationships may compromise the development of mentalizing capacities, rendering individuals more vulnerable to transient collapses of reflective functioning in emotionally charged situations [12,27]. This interpretation is consistent with the findings of Quek et al. (2018), which support the role of mentalization in borderline psychopathology during adolescence and suggest that mentalizing difficulties are already associated with borderline manifestations during early developmental periods [68].

Finally, the associations observed with positive urgency also deserve attention, although they appeared more modest than those identified for negative urgency. They suggest that mentalizing difficulties are not exclusively involved in impulsive responses associated with negative affect but may also play a role in contexts characterized by intense emotional activation regardless of emotional valence. However, given their lower robustness, more modest effect sizes, model diagnostics indicating potential overfitting in the BPD subgroup, and the limited literature specifically linking mentalizing difficulties to positive urgency, these findings should be considered exploratory and require replication in independent clinical samples. Nevertheless, the available evidence suggests that mentalizing difficulties are associated with diverse forms of emotion-related impulsivity, including addictive, aggressive, and risk-taking behaviors, although these observations derive primarily from non-clinical samples or populations presenting other forms of psychopathology [69,70].

An additional consideration concerns the psychometric properties of the RFQ, with some authors suggesting that this instrument primarily measures a dimension of hypomentalizing and that certain items may reflect, at least partially, characteristics closely related to emotional lability or impulsivity [36]. Consequently, some of the observed associations may be attributable to overlapping between the constructs assessed. Despite this limitation, the persistence of the effects after adjustment for alexithymia, anxiety–depressive symptomatology, and borderline diagnosis argues in favor of a specific association of mentalizing difficulties with emotion-related impulsivity.

The coefficients observed within the BPD subgroup were generally more modest than those found in the overall sample. Several explanations may account for this pattern. A restriction of variance is likely in a population already characterized by elevated levels of emotion-related impulsivity and mentalizing difficulties. The clinical heterogeneity of BPD may also attenuate certain associations, as multiple mechanisms, including early trauma, attachment disturbances, dissociative phenomena, and identity difficulties, may simultaneously contribute to the expression of impulsivity. Finally, the relatively limited size of the BPD subgroup (*n* = 45) may have reduced the statistical power available to detect associations of smaller magnitude. Accordingly, non-significant findings within the BPD subgroup should not be interpreted as evidence of absence. Given the modest sample size and the possibility of restricted variability within a clinically severe group, smaller effects may have remained undetected.

### 4.6. Strengths and Limitations

This study has several strengths. First, it builds upon an ongoing research program conducted within the same cohort of young individuals aged 16 to 25 years, in which we have successively examined emotion-related impulsivity, emotional dysregulation, alexithymia, and mentalizing difficulties in BPD. This cumulative approach enables the findings of the present study to be interpreted within a broader, integrated understanding of the psychopathological mechanisms underlying the emergence of borderline pathology during this particularly vulnerable developmental period.

Furthermore, the variables under investigation were assessed using validated instruments that are widely employed in international literature. Statistical analyses also represent a methodological strength of this study. The associations between mentalizing difficulties and the emotional urgency dimensions were examined using multivariate models adjusted for several clinically relevant variables, including alexithymia, depressive symptomatology, trait anxiety, and borderline diagnosis when applicable. The use of robust standard errors allowed us to account for potential heteroscedasticity in the data. In addition, the application of a correction for multiple comparisons strengthens the robustness of the reported findings, even though it may have reduced the ability to detect associations of smaller magnitude. Finally, the persistence of the associations between uncertainty regarding mental states and the emotional urgency dimensions within the BPD subgroup itself suggests that mentalizing difficulties provide clinically meaningful information beyond diagnostic status alone. The joint examination of alexithymia and mentalization also constitutes an original contribution of this study, allowing for a more precise delineation of their respective roles in emotion-related impulsivity in BPD.

Nevertheless, this study also presents several limitations. First, its cross-sectional design does not permit determination of the directionality of the observed associations or causal inferences regarding the relationships between mentalizing difficulties and emotion-related impulsivity. It therefore remains possible that mentalizing difficulties increase the propensity to act impulsively under the influence of intense emotions, that emotion-related impulsivity secondarily impairs reflective capacities, or that both phenomena are influenced by common underlying psychopathological mechanisms.

Second, all variables were assessed using self-report measures. All principal variables—including RFQu, DERS, UPPS-P, TAS-20, BDI-II, and STAI-T—were assessed using self-report questionnaires administered concurrently. This reliance on a single method of assessment raises the possibility that shared method variance, such as common response styles, general negative affectivity, or introspective accuracy, may have inflated the observed associations, including the central association between RFQu and negative urgency. This limitation appears particularly important with respect to mentalization, insofar as the RFQ relies on individuals’ self-evaluation of their reflective capacities even though these individuals may themselves experience difficulties identifying, understanding, and evaluating their own mental states. This conceptual limitation, far from trivial, has been emphasized in recent critiques of this instrument [36]. Moreover, RFQu constitutes a self-reported indicator of uncertainty regarding mental states rather than a direct measure of mentalizing performance; consequently, its interpretation as reflecting hypomentalizing should remain cautious. The absence of clinician-rated or behavioral/performance-based measures of mentalizing, such as structured interviews or observational tasks (e.g., the Movie for the Assessment of Social Cognition, MASC), further limits our ability to disentangle self-reported mentalizing beliefs from actual mentalizing capacity, and prevents any triangulation that could help clarify the extent to which the present findings reflect shared method variance rather than substantive associations.

In addition, RFQu scores were derived from the extraction of the corresponding eight items from the long version of the Reflective Functioning Questionnaire (RFQ-54) rather than from the independent administration of the RFQ-8. Although this strategy is consistent with the scoring procedures proposed in the initial validation studies of the RFQ [35], we emphasize this as an explicit limitation given the central role of RFQu in the present findings: item-level psychometric properties (e.g., item difficulty, discrimination, or response distribution) derived within the longer RFQ-54 questionnaire context may differ from those obtained when the RFQ-8 is administered as a standalone instrument, and this cannot be ruled out as a contributing factor to the observed associations.

Third, a further limitation concerns the psychometric status of the RFQ itself. Recent large-scale psychometric evaluations have raised broader concerns than those already noted regarding the RFQc subscale. Using the standalone 8-item RFQ administered independently across three large samples, Müller et al. found no consistent evidence for the originally proposed bidimensional structure of the instrument, suggesting instead that it may capture a single underlying dimension, and showed that the double-scoring procedure used to derive RFQc and RFQu introduces a mathematical, rather than substantive, negative association between the two subscales [36]. More critically, using preregistered commonality analyses, the same authors found that associations between the RFQ and indicators of personality dysfunction were substantially inflated by item content overlapping with emotional lability and negative urgency rather than mentalizing per se, with certain items (including one closely resembling standard negative urgency items) driving this confound [36]. Given that the present study’s central finding concerns the association between RFQu and negative urgency specifically, this item-level content overlap cannot be excluded as a partial contributor to the observed association, independently of any genuine link between mentalizing and impulsivity. Future studies should therefore aim to replicate these findings using alternative or complementary measures of mentalizing with more clearly differentiated item content, such as performance-based tasks or self-report instruments developed to minimize conceptual overlap with impulsivity and affective instability.

Fourth, the relatively limited size of the BPD subgroup (*n* = 45) invites caution in the interpretation of the multivariate models conducted within this subgroup. Although the observed associations appeared robust, the sample size may have limited statistical power, increased the instability of the estimates, and reduced the generalizability of the findings. In addition, regression models conducted within the BPD subgroup should be considered exploratory given the limited number of participants relative to the number of covariates included. Furthermore, the globally elevated levels of emotional dysregulation, emotion-related impulsivity, and mentalizing difficulties observed in the BPD group may have resulted in a restriction of variance, potentially attenuating certain associations. This hypothesis may partly explain why several correlations observed in the overall sample no longer remained significant after correction for multiple comparisons within the BPD subgroup. Therefore, non-significant findings within the BPD subgroup should not be interpreted as evidence of absence, as smaller effects may have remained undetected. Relatedly, the ratio of observations to predictors in the BPD subgroup analyses (*n* = 45 for four covariates, approximately 11:1) is at the lower end of commonly recommended thresholds, raising the possibility of model overfitting. For the quantile regression models comparing mentalization scores between controls and individuals with BPD, multicollinearity diagnostics indicated no evidence of collinearity among covariates (mean variance inflation factor = 2.28 [<5]; condition index = 9.20 [<10]), and a training–test sample comparison indicated no substantial overfitting (mean absolute error increase of 19.0–22.3% from training to test samples); residual diagnostics revealed no systematic pattern or influential outliers. For this adjusted comparison model, the 95% confidence interval for RFQuHypo total was relatively narrow (b = 4.7, 95% CI [1.31, 8.03], pseudo-R^2^ = 0.305), supporting the precision of this estimate, whereas the corresponding estimate for RFQcHyper total was imprecise and non-significant (b = 2.0, 95% CI [−1.32, 5.32], *p* = 0.236, pseudo-R^2^ = 0.082), consistent with the weaker and less consistent evidence for hypermentalizing reported throughout this study. These diagnostics, however, pertain to the larger adjusted comparison model (*n* = 166) rather than to the smaller BPD-subgroup linear regressions predicting negative and positive urgency (*n* = 45). For these four linear regression models (Table 8, Table 9, Table 10 and Table 11), multicollinearity diagnostics indicated no evidence of collinearity (mean VIF ranging from 2.400 to 2.460, all <5), and Leave-One-Out Cross-Validation (LOOCV) was conducted to assess overfitting given that the limited subgroup sample size precluded a training–test split. For the model predicting negative urgency within the BPD subgroup (Table 9), LOOCV indicated no substantial overfitting (RMSE increase of 5.26% [<10%]; adjusted R^2^ decrease of 18.25% [<20%]), supporting the stability of this model. For the model predicting positive urgency within the BPD subgroup (Table 11), however, LOOCV indicated potential overfitting (RMSE increase of 9.81%, approaching the 10% threshold; adjusted R^2^ decrease of 65.24%, exceeding the 20% threshold), indicating that this model’s estimates should be interpreted with particular caution. The fact that RFQu remained significantly associated with negative urgency despite the modest subgroup size, with a relatively narrow bias-corrected bootstrap 95% confidence interval (95% CI [0.21, 0.59]) and no evidence of overfitting on cross-validation, confirms the robustness of this specific finding; in contrast, the corresponding association with positive urgency did not reach statistical significance once bias-corrected bootstrap confidence intervals were used (95% CI [−0.04, 0.44]), and the LOOCV diagnostics further indicate that this secondary, non-significant finding is the least statistically stable of the four models tested. Although our findings appear promising despite the limited size of our BPD sample, they should be interpreted as preliminary; larger-scale studies are needed to improve statistical power and confirm these results.

Finally, our sample consisted predominantly of women (93.3% of the BPD group), thereby limiting the generalizability of the findings to men with BPD. This limitation may not affect all reported associations equally. In a recent study of young individuals with mood disorders, Di Benedetto et al. found gender to be a significant predictor of alexithymia (TAS-20) and global emotion dysregulation (DERS), with women scoring higher on both, as well as of the Lack of Premeditation and Lack of Perseverance dimensions of the UPPS-P, but found no significant gender effect on either negative or positive urgency [71]. If a similar pattern applies to BPD, the central association reported in the present study—between RFQu and negative urgency—may be relatively more robust to the sample’s gender imbalance, whereas associations involving total alexithymia (TAS-20) and global emotion dysregulation (DERS) could be more sensitive to the predominantly female composition of the sample. This distinction should nonetheless be interpreted with caution, as gender effects on these constructs may differ between mood disorders and BPD populations.

Lastly, the absence of a psychiatric comparison group constitutes another important limitation. Comparisons with patients presenting other psychiatric disorders frequently associated with emotional dysregulation and impulsivity, such as mood disorders, substance use disorders, or ADHD, would have enabled a more precise assessment of the specificity of the associations observed in BPD. This point is reinforced by recent meta-analytic evidence confirming that emotion dysregulation, while most pronounced in BPD, is nonetheless a transdiagnostic feature present with large effect sizes across a broad range of psychiatric disorders [52]. Without a psychiatric comparison group, it is therefore not possible to determine whether the elevated RFQu observed in our BPD sample reflects a mentalizing vulnerability specific to BPD or a characteristic shared with other clinical conditions marked by severe emotion dysregulation. Moreover, BPD is a clinically heterogeneous disorder, and other mechanisms potentially involved in emotion-related impulsivity, such as early trauma, attachment disturbances, dissociative phenomena, or identity-related difficulties, were not considered in the present study. Mentalizing difficulties should therefore be regarded as one of several factors potentially associated with the emotion-related impulsivity observed in BPD rather than as its sole determinant. Future longitudinal studies involving larger samples, multimodal assessments of mentalization, and psychiatric comparison groups are therefore needed to clarify the role of mentalizing difficulties in the emotion-related impulsivity associated with borderline personality disorder.

### 4.7. Theoretical and Clinical Implications and Future Research Directions

The findings of the present study do not necessarily support the hypothesis of a global and stable deficit in mentalization among individuals with BPD. Rather, they appear more consistent with contemporary conceptualizations of mentalization, according to which the difficulties observed in BPD reflect a context-dependent vulnerability that manifests preferentially in situations characterized by heightened emotional and interpersonal activation. From this perspective, mentalizing capacities are not continuously impaired but instead become particularly fragile when attachment systems and stress-response systems are strongly activated [57]. In a convergent manner, Petersen et al. demonstrated that individuals with BPD do not necessarily exhibit uniform impairments in mentalizing capacities, with some abilities appearing relatively preserved while difficulties emerge in situations requiring more complex mental state inferences [72]. These observations suggest that the mentalizing difficulties observed in BPD may reflect a context-dependent fragility of reflective functioning rather than a generalized deficit.

Given the cross-sectional design of the present study, the clinical implications developed below (including the proposed complementarity between MBT and DBT and the relevance of a clinical staging framework) should be regarded as exploratory and hypothesis-generating rather than as conclusions directly supported by the data. From a clinical perspective, the finding that uncertainty regarding mental states remained associated with the emotional urgency dimensions independently of alexithymia and anxiety–depressive distress suggests that mentalizing difficulties may constitute a relevant therapeutic target beyond difficulties in emotional identification alone. These findings concern not only young adults with BPD but also the overall sample, in which mentalizing difficulties were associated with emotional dysregulation and the emotional dimensions of impulsivity. This observation suggests that mentalizing capacities may represent a transdiagnostic therapeutic target of interest among young adults experiencing difficulties in emotion regulation, regardless of the presence of a formal borderline diagnosis.

Among participants with BPD, the persistence of the associations between RFQu and negative urgency within the BPD group itself suggests that the assessment of mentalization provides information that is not redundant with the diagnosis. Mentalizing capacities may therefore contribute to a more refined characterization of the clinical heterogeneity observed within a diagnostically homogeneous population and help identify patients who are particularly prone to reacting impulsively under the influence of intense negative emotions. Within this framework, mentalizing difficulties may constitute a therapeutic target complementary to interventions already employed in BPD.

These findings do not support the superiority of any specific psychotherapeutic approach. Nevertheless, they suggest that mentalization-based interventions and interventions derived from Dialectical Behavior Therapy (DBT) may act on different yet potentially complementary processes. Whereas DBT primarily aims to develop skills related to mindfulness, distress tolerance, emotion regulation, and behavioral control, Mentalization-Based Treatment (MBT) more specifically seeks to restore the capacity to understand one’s own and others’ mental states in contexts of heightened emotional and relational activation [73]. It is noteworthy that the concept of Wise Mind, proposed by Linehan, which refers to the integration of emotional and rational information to achieve a more balanced understanding of internal experience, shares certain similarities with reflective functioning without being equivalent to it. Whereas Wise Mind primarily emphasizes the integration of different modes of processing subjective experience, mentalization involves the explicit elaboration of one’s own and others’ mental states [8]. The joint study of these constructs may contribute to a better understanding of the therapeutic mechanisms involved in current interventions for patients with BPD.

This interpretation is consistent with the available evidence regarding MBT. In their systematic review addressing the therapeutic management of impulsivity in BPD, Mungo et al. (2021) emphasized that psychotherapy remains the treatment of choice and identified MBT as one of the approaches showing promising effects on the impulsive manifestations of the disorder [73]. Moreover, De Meulemeester et al. (2020) demonstrated that improvements in mentalizing capacities during treatment were associated with reductions in symptomatic distress among patients with BPD, suggesting that changes in mentalization may contribute to therapeutic change without necessarily constituting its sole explanatory mechanism [74].

These findings also appear particularly relevant considering contemporary approaches to early intervention and clinical staging models of BPD. The identification of mentalizing difficulties associated with emotion-related impulsivity as early as late adolescence and emerging adulthood suggests that these vulnerabilities may constitute targets for intervention before the consolidation of more severe psychopathological trajectories. Clinical staging models propose tailoring interventions according to the severity and developmental stage of borderline manifestations, with particular attention devoted to young individuals exhibiting emerging borderline traits or high-risk profiles [75]. Within this perspective, the assessment of mentalizing capacities may contribute to a more precise characterization of vulnerable youth and facilitate the early targeting of interventions toward the most relevant mechanisms. This proposition is consistent with the work of Quek et al. (2018), emphasizing the importance of mentalization processes in adolescent borderline psychopathology [68], as well as with our previous findings highlighting the central role of emotion-related impulsivity in emerging BPD [9].

Finally, despite the reservations expressed regarding certain psychometric properties of the RFQ [36], the assessment of RFQu may hold potential clinical value as an indicator of a specific therapeutic target. Elevated levels of uncertainty regarding mental states have indeed been associated with borderline psychopathology and have been shown to discriminate patients with BPD from healthy controls [54]. In this context, RFQu may contribute to the identification of patients who are particularly vulnerable to failures of mentalization in situations of emotional distress. However, further research is required to clarify the implications of these observations. Future studies should determine whether the associations identified in the present study are specific to BPD or instead reflect broader transdiagnostic mechanisms shared with other psychiatric populations characterized by pronounced emotion-related impulsivity. Longitudinal investigations would also help determine whether mentalizing difficulties contribute to the persistence or worsening of borderline manifestations over the course of development and whether they may assist in identifying young individuals following more severe psychopathological trajectories within a clinical staging framework [75]. Finally, future studies would benefit from diversifying methods of assessing mentalization by combining self-report measures, performance-based measures such as the MASC and approaches based on clinical interviews. Joint exploration of the relationships among mentalization, Wise Mind, and emotion-related impulsivity may also contribute to a better understanding of the common or complementary therapeutic mechanisms involved in mentalization-based approaches and DBT.

## 5. Conclusions

The present study aimed to examine the relationships among mentalizing difficulties, emotional dysregulation, and emotion-related impulsivity in older adolescents and young adults with borderline personality disorder. Uncertainty regarding mental states was associated with more severe emotional dysregulation, particularly with difficulties in maintaining behavioral control under emotional distress, as well as with the emotional dimensions of impulsivity, most notably negative urgency.

Multivariate analyses further suggest that the association between uncertainty regarding mental states and emotion-related impulsivity persists beyond the effects of alexithymia, anxiety and depressive symptoms, and, in the overall sample, borderline status. These findings indicate that mentalizing difficulties may account for additional variance in the manifestations of emotion-related impulsivity observed among young individuals with BPD, beyond the contribution of alexithymic difficulties and generalized psychopathological distress as assessed in the present study.

From a broader perspective, this study supports the value of a developmental and mechanism-focused approach to the understanding of emerging BPD. Mentalizing difficulties appear to represent a potentially relevant process through which the emotional, interpersonal, and behavioral dimensions of the disorder may be integrated. Their early identification may contribute to a more precise characterization of vulnerability profiles among young individuals presenting borderline manifestations and may help guide interventions toward appropriate therapeutic targets.

Finally, longitudinal, multimodal studies including psychiatric comparison groups remain necessary to clarify the temporal sequence and specificity of these associations; to examine more closely the interactions among mentalization, emotion regulation, and impulsivity; and to better understand whether strengthening mentalizing capacities may contribute to the prevention or attenuation of the most severe psychopathological trajectories associated with BPD.

## Figures and Tables

**Table 1:** Sociodemographic and General Clinical Characteristics of Participants (*n* = 166).

Variables	Categories	%	Controls (*n* = 121)	BPD Subjects (*n* = 45)	*p*-Value Chi^2^
Gender	Male (*n* = 35) Female (*n* = 131)	21.1% 78.9%	26.5% 73.5%	6.7% 93.3%	0.005
Student	No (*n* = 11) Yes (*n* = 155)	6.6% 93.4%	5.8% 94.2%	8.9% 91.1%	0.475
Somatic and/or surgical history	No (*n* = 63) Yes (*n* = 103)	38.0% 62.0%	37.2% 62.8%	40.0% 60.0%	0.740
Psychiatric history	No (*n* = 143) Yes (*n* = 23)	86.1% 13.9%	97.5% 2.5%	55.6% 44.4%	<0.001
Psychiatric comorbidities	0 (*n* = 133) 1 (*n* = 15) ≥2 (*n* = 18)	80.1% 9.0% 10.9%	100.0% 0.0% 0.0%	26.7% 33.3% 40.0%	<0.001
Alcohol	No (*n* = 49) Occasional (*n* = 92) Binge drinking (*n* = 25)	29.5% 55.4% 15.1%	33.1% 51.2% 15.7%	20.0% 66.7% 13.3%	0.180
Drugs	No (*n* = 139) Yes (*n* = 27)	83.7% 16.3%	95.0% 5.0%	53.3% 46.7%	<0.001
Family psychiatric history	No (*n* = 107) Yes (*n* = 59)	64.5% 35.5%	83.5% 16.5%	13.3% 86.7%	<0.001
History of suicide attempts	No (*n* = 122) Yes (*n* = 44)	73.5% 26.5%	92.6% 7.4%	22.2% 77.8%	<0.001
Past psychological follow-up	No (*n* = 117) Yes (*n* = 49)	70.5% 29.5%	89.3% 10.7%	20.0% 80.0%	<0.001
Actual psychological follow-up	No (*n* = 121) Yes (*n* = 45)	72.9% 27.1%	100.0% 0.0%	0.0% 100.0%	<0.001
Psychotropic treatment	No (*n* = 134) Yes (*n* = 32)	80.7% 19.3%	100.0% 0.0%	28.9% 71.1%	<0.001
Educational level	Primary education (*n* = 96) Secondary or higher education (*n* = 70)	57.8% 42.2%	52.9% 47.1%	71.1% 28.9%	0.035
	**Median** **(P25–P75)**				**Wilcoxon test**
Age (years)	18 (17–21)		18 (17–22)	17 (16–21)	0.069
Number of suicide attempts	0 (0–1)		0 (0–0)	2 (1–5)	<0.001
Number of substances	0 (0–0)		0 (0–0)	0 (0–1)	<0.001

**Table 2:** Psychometric Characteristics of the Sample.

Variables	Median (P25–P75)	Controls (*n* = 121)	BPD Subjects (*n* = 45)	Wilcoxon Test
RFQc	4 (2–7)	4 (2–8)	4 (3–6)	0.828
RFQu	5 (3–9)	4 (2–7)	11 (7–13)	<0.001
BDI-II	15 (9–28)	12 (8–19)	33 (19–45)	<0.001
STAI-T	50 (40–61)	46 (37–53)	66 (58–72)	<0.001
UPPS urgencies negative	11 (8–13)	10 (8–12)	14 (11–15)	<0.001
UPPS urgencies positive	12 (10–14)	12 (10–13)	14 (12–16)	<0.001
UPPS lacks premeditation	9 (7–11)	8 (6–10)	11 (9–13)	<0.001
UPPS lacks perseverance	8 (6–11)	7 (5–9)	11 (9–12)	<0.001
UPPS seeks sensation	12 (9–14)	11 (9–14)	13 (9–15)	0.103
TAS DIF	23 (16–27)	20 (15–26)	25 (23–29)	<0.001
TAS DDF	16 (13–19)	16 (13–18)	18 (15–19)	0.072
TAS EOT	27 (25–29)	27 (25–30)	26 (24–27)	0.014
TAS total	66 (58–72)	63 (57–71)	68 (63–74)	0.022
DERS Non-Acceptance	13 (9–22)	12 (8–17)	22 (15–26)	<0.001
DERS Goals	20 (14–23)	18 (13–22)	23 (20–25)	<0.001
DERS Impulse	15 (10–22)	13 (9–18)	23 (18–28)	<0.001
DERS Awareness	17 (13–22)	15 (12–19)	22 (18–25)	<0.001
DERS Clarity	14 (9–18)	11 (9–16)	18 (15–20)	<0.001
DERS Strategies	24 (17–32)	21 (15–28)	33 (27–36)	<0.001
DERS total	105 (80–131)	93 (73–114)	138 (124–148)	<0.001

**Table 8:** Linear regression analyses for UPPS urgencies negative in whole sample (*n* = 166).

	Coefficient (β)	Bootstrap SE	z	*p*-Value	95% CI
Constant	7.38	1.42	5.21	<0.001	4.64 to 10.13
RFQu	0.42	0.07	6.39	<0.001	0.30 to 0.56
TAS total	0.01	0.02	0.27	0.790	−0.04 to 0.06
BDI-II	−0.04	0.03	−1.53	0.126	−0.08 to 0.02
STAI-T	0.02	0.03	0.71	0.480	−0.03 to 0.07
BPD diagnosis	0.85	0.66	1.29	0.197	−0.49 to 2.10
	**R^2^ = 0.3995**				

Multicollinearity diagnostics: absence of multicollinearity (mean Variance Inflation Factors: 2.460 [<5]). Overfitting diagnostics (Leave-One-Out Cross-Validation): no substantial overfitting (RMSE 2.5122 and RMSE_LOOCV 2.5623: increase of 1.99% [<10%]—R^2^ 0.3995 and adjusted R^2^ 0.3807: decrease of 4.71% [<20%]). Residuals diagnostics: no systematic pattern or influential outliers.

**Table 9:** Linear regression analyses for UPPS urgencies negative in BPD subjects (*n* = 45).

	Coefficient (β)	Bootstrap SE	z	*p*-Value	95% CI
Constant	13.65	3.14	4.35	<0.001	7.26 to 19.55
RFQu	0.41	0.10	4.13	<0.001	0.21 to 0.59
TAS total	−0.06	0.04	−1.35	0.175	−0.17 to 0.01
BDI-II	−0.05	0.04	−1.43	0.151	−0.13 to 0.02
STAI-T	0.01	0.05	0.28	0.777	−0.08 to 0.11
	**R^2^ = 0.3540**				

Multicollinearity diagnostics: absence of multicollinearity (mean Variance Inflation Factors: 2.400 [<5]). Overfitting diagnostics (Leave-One-Out Cross-Validation): no substantial overfitting (RMSE 2.5855 and RMSE_LOOCV 2.7216: increase of 5.26% [<10%]—R^2^ 0.3540 and adjusted R^2^ 0.2894: decrease of 18.25% [<20%]). Residuals diagnostics: no systematic pattern or influential outliers.

**Table 10:** Linear regression analyses for UPPS urgencies positive in whole sample (*n* = 166).

	Coefficient (β)	Bootstrap SE	z	*p*-Value	95% CI
Constant	8.65	1.33	6.51	<0.001	6.20 to 11.36
RFQu	0.12	0.08	1.63	0.104	−0.02 to 0.27
TAS total	0.03	0.02	1.34	0.180	−0.01 to 0.08
BDI	0.01	0.03	0.25	0.803	−0.05 to 0.06
STAI-T	0.01	0.03	0.32	0.749	−0.04 to 0.07
BPD diagnosis	0.57	0.63	0.90	0.370	−0.78 to 1.68
	**R^2^ = 0.1633**				

Multicollinearity diagnostics: absence of multicollinearity (mean Variance Inflation Factors: 2.460 [<5]). Overfitting diagnostics (Leave-One-Out Cross-Validation): no substantial overfitting (RMSE 2.4753 and RMSE_LOOCV 2.5388: increase of 2.57% [<10%]—R^2^ 0.1633 and adjusted R^2^ 0.1371: decrease of 16.04% [<20%]). Residuals diagnostics: no systematic pattern or influential outliers.

**Table 11:** Linear regression analyses for UPPS urgencies positive in BPD subjects (*n* = 45).

	Coefficient (β)	Bootstrap SE	z	*p*-Value	95% CI
Constant	10.87	2.74	3.97	<0.001	4.84 to 15.99
RFQu	0.21	0.12	1.71	0.087	−0.04 to 0.44
TAS total	0.01	0.04	0.25	0.803	−0.06 to 0.17
BDI	0.02	0.04	0.44	0.661	−0.06 to 0.13
STAI-T	−0.02	0.06	−0.32	0.752	−0.13 to 0.07
	**R^2^ = 0.1329**				

Multicollinearity diagnostics: absence of multicollinearity (mean Variance Inflation Factors: 2.400 [<5]). Overfitting diagnostics (Leave-One-Out Cross-Validation): potential overfitting (RMSE 2.6465 and RMSE_LOOCV 2.9062: increase of 9.81% [<10%]—R^2^ 0.1329 and adjusted R^2^ 0.0462: decrease of 65.24% [failure to meet the threshold < 20%]). Residuals diagnostics: no systematic pattern or influential outliers.

## Data Availability

The corresponding author may provide the data for this study upon reasonable request.

## Abbreviations

The following abbreviations are used in this manuscript:

ADHD	Attention-Deficit/Hyperactivity Disorder
BDI-II	Beck Depression Inventory—Second Edition
BPD	Borderline Personality Disorder
DBT	Dialectical Behavior Therapy
DDF	Difficulty Describing Feelings
DERS	Difficulties in Emotion Regulation Scale
DIF	Difficulty Identifying Feelings
DIB-R	Diagnostic Interview for Borderlines—Revised
DSM-5	Diagnostic and Statistical Manual of Mental Disorders, 5th Edition
EOT	Externally Oriented Thinking
MASC	Movie for the Assessment of Social Cognition
MBT	Mentalization-Based Treatment
MentS	Mentalization Scale
RFQ	Reflective Functioning Questionnaire
RFQ-8	Reflective Functioning Questionnaire—8-item short version
RFQ-54	Reflective Functioning Questionnaire—54-item long version
RFQc	Reflective Functioning Questionnaire—Certainty subscale
RFQu	Reflective Functioning Questionnaire—Uncertainty subscale
SE	Standard Error
STAI-T	State-Trait Anxiety Inventory—Trait subscale
TAS-20	Toronto Alexithymia Scale—20 items
TAS-DDF	Toronto Alexithymia Scale—Difficulty Describing Feelings subscale
TAS-DIF	Toronto Alexithymia Scale—Difficulty Identifying Feelings subscale
TAS-EOT	Toronto Alexithymia Scale—Externally Oriented Thinking subscale
UPPS-P	Urgency, Premeditation, Perseverance, Sensation Seeking, Positive Urgency impulsivity scale
